# Deep Learning in Diverse Intelligent Sensor Based Systems

**DOI:** 10.3390/s23010062

**Published:** 2022-12-21

**Authors:** Yanming Zhu, Min Wang, Xuefei Yin, Jue Zhang, Erik Meijering, Jiankun Hu

**Affiliations:** 1 School of Computer Science and Engineering, University of New South Wales, Sydney, NSW 2052, Australia; 2 School of Engineering and Information Technology, University of New South Wales, Canberra, ACT 2612, Australia

**Keywords:** deep learning, computer vision, biomedical imaging, biometrics, remote sensing, cybersecurity, Internet of Things, natural language processing, audio and speech processing, control system and robotics, information system, food, agriculture, chemistry

## Abstract

Deep learning has become a predominant method for solving data analysis problems in virtually all fields of science and engineering. The increasing complexity and the large volume of data collected by diverse sensor systems have spurred the development of deep learning methods and have fundamentally transformed the way the data are acquired, processed, analyzed, and interpreted. With the rapid development of deep learning technology and its ever-increasing range of successful applications across diverse sensor systems, there is an urgent need to provide a comprehensive investigation of deep learning in this domain from a holistic view. This survey paper aims to contribute to this by systematically investigating deep learning models/methods and their applications across diverse sensor systems. It also provides a comprehensive summary of deep learning implementation tips and links to tutorials, open-source codes, and pretrained models, which can serve as an excellent self-contained reference for deep learning practitioners and those seeking to innovate deep learning in this space. In addition, this paper provides insights into research topics in diverse sensor systems where deep learning has not yet been well-developed, and highlights challenges and future opportunities. This survey serves as a catalyst to accelerate the application and transformation of deep learning in diverse sensor systems.

## 1. Introduction

In recent years, driven by the rapid increase in available data and computational resources, deep learning has achieved extraordinary advances and almost become the de-facto standard approach in virtually all fields of science and engineering. Essentially, deep learning is a part of the field of machine learning, a subfield of artificial intelligence (AI) concerned with learning data representations using computational methods. In traditional machine learning algorithms, manually choosing features and a classifier is needed, while in a deep learning algorithm, the features are extracted automatically by the algorithm through learning from its own errors. It is this automatic feature extraction that distinguishes deep learning from the field of machine learning.

Neural networks make up the backbone of deep learning algorithms. A neural network aims to learn nonlinear maps between inputs and outputs through its elementary computational cells (also called “neurons”). It is the number of layers (also called depth) of neural networks that distinguishes a shallow network from a Deep Neural Network (DNN). Typically, a network must have more than three layers to be considered a DNN. Deep networks learn representations of the data in a hierarchical manner to simulate the mechanism of the human brain in extracting information from given data.

The increasing complexity and the large volume of data collected by diverse sensor systems have brought about significant developments in deep learning, which have fundamentally transformed the way the data are acquired, processed, analyzed, and interpreted. Therefore, in this paper, we provide a comprehensive investigation of deep learning in diverse intelligent sensor based systems, covering fundamentals of deep learning models and methods, deep learning techniques for fundamental tasks in individual sensor systems, insights of reformulation of these fundamental tasks for broader applications in diverse intelligent sensor based systems, and challenges of breaking through the bottleneck of current deep learning approaches in exploring the full potential of deep learning. We searched Google Scholar (GS) and Web of Science (WOS) with the keywords deep learning (DL) and sensor. This resulted in 16,100 articles from 2020. We further selected, based on the top journals and conferences, around 150 most relevant papers for careful inspection, and traced some further relevant references from there. From these, we observed that existing relevant surveys [1,2,3,4] have one or more of the following limitations: (1) touching only a small subset of topics in individual domains, (2) lacking an overview of common techniques/algorithms from different domains, and (3) lacking a holistic view based on the individual domains of diverse intelligent sensor based systems. This survey aims to be a catalyst for accelerating the application and transformation of deep learning across diverse intelligent sensor based systems.

The contributions of this paper can be summarized as follows.

- This is the first paper to provide a comprehensive investigation of deep learning in diverse sensor systems from the perspective, in a holistic view, of different data modalities across different intelligent sensor based systems and application domains.
- This paper presents the fundamentals of deep learning and the most widely used deep learning models and methods in a concise and high-level way, which would be very useful for people to get a quick start in the field.
- This paper provides a comprehensive summary of deep learning implementation tips and links to tutorials, open-source codes, and pretrained models, which can serve as an excellent self-contained reference for deep learning practitioners and researchers. This is a unique feature that makes it distinguishable from existing literature survey papers.
- This paper identifies the fundamental tasks in individual intelligent sensor based systems and provides insights to reformulation of these task for broader applications for those seeking to innovate deep learning in diverse sensor systems.
- This paper provides insights into research topics where deep learning has not yet been well-developed, and highlights the challenges and future directions of deep learning in diverse intelligent sensor based systems.

## 2. Deep Learning Basics

### 2.1. History of Deep Neural Networks

The origin of DNNs can be traced back to 1943, when McCulloch and Pitts proposed the first artificial neural network [5]. Since then, deep learning has grown gradually and achieved a few significant milestones in its development. One of them worth mentioning is Rosenblatt’s “perceptron” introduced in 1958. It demonstrated that a perceptron will converge when what they are trying to learn can be represented [6]. However, such a model has obvious limitations, and multilayer perceptrons are required by complex tasks, but at that time, it was not clear how to train these models. Subsequently, deep learning encountered its first winter.

Until 1985, Hinton et al. proposed the back-propagation algorithm, which has greatly stimulated the development of this field [7]. At almost the same period, the “neocogitron” which inspired the Convolutional Neural Networks (CNNs), the Recurrent Neural Networks (RNNs), and the DNNs were proposed [8,9,10]. However, due to the limitation of hardware, these models were hard to use for handling large data, and thus the development of deep learning was trapped again.

By 2006, Hinton and others solved the training problem of DNNs by using a layer-wise pretraining framework, which greatly revitalized the field [11,12]. At the same time, algorithms for training deep AutoEncoders (AEs), and other deep architectures were proposed [13], which allowed deep learning to develop at an exponential rate. From then, a variety of deep learning methods increasingly emerged, including Deep Belief Networks (DBNs), Restricted Boltzmann Machines (RBMs), CNNs, Generative Adversarial Networks (GANs), Graph Neural Networks (GNNs), and so on.

In recent years, two astounding deep learning applications made a global splash and shocked the world. One is AlphaGo, which defeated the world champion Go players using deep learning with the support of abundant hardware resources (https://www.deepmind.com/research/highlighted-research/alphago accessed on 2 November 2022). Another is AlphaFold, which solved the 50-year-old challenging protein folding problem. These further stimulated the rapid development of deep learning in various domains. Nowadays, with the advancements in Graphics Processing Units (GPUs) and High-Performance Computing (HPC), deep learning has become one of the most efficient tools with outstanding performance in almost every domain.

### 2.2. Fundamentals of Deep Neural Networks

**Figure 1:**
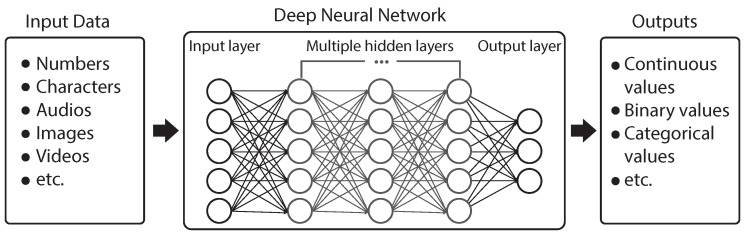
Diagram of a DNN.

DNNs try to mimic the way biological neurons send signals to each other through numerous neurons (also called nodes). Generally, the architecture of a DNN consists of multiple neuron layers including an input layer, an output layer, and one or many hidden layers [14] (Figure 1). Each neuron is connected to another neuron to pass information. The input to a DNN can be numbers, characters, audios, images, etc., which are broken down into bits of binary data that a computer can process. The output can be continuous values, binary values, or categorical values, depending on the tasks. A DNN relies on training data to learn and improve its accuracy over time. During the learning, if it cannot accurately recognize a particular pattern for a given task, an algorithm would adjust its weights until it determines the correct mathematical manipulation to fully process the data [13].

#### 2.2.1. Neuron Perception

**Figure 2:**
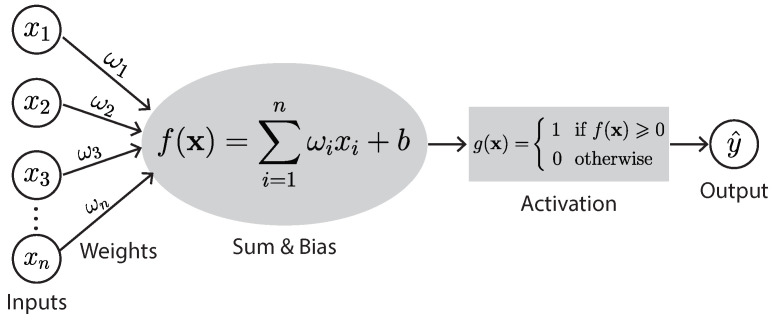
Diagram of the perception of a neuron.

A neuron multiplies each of its inputs by an associated weight and then sums these weighted inputs and adds a predetermined number called the bias (Figure 2). The neuron is activated if its output is above a specified threshold and will pass its output to the next layer of the DNN. That is, in a DNN, neurons in each layer get inputs from the previous layer, learn representations, and then pass the information to the next layer. Each successive layer of a DNN uses the output of the previous layer for its input. This way, a DNN produces an output at the end.

#### 2.2.2. Activation Functions

The activation function is an important aspect of a DNN. It defines the output of a node given inputs and is mainly used to generate a nonlinear relationships between the input and the output. Currently, there are 10 types of nonlinear activation functions (Table 1). Here, we elaborate on four most popular activation functions, namely sigmoid, tanh, ReLU, and leaky ReLu, describe their application scenarios, and analyze their pros and cons.

The sigmoid activation function is one of the most widely used activation functions. It takes an arbitrary value as input and outputs a value between 0 and 1. The larger the input, the closer the output value is to 1. This function is differentiable and provides a smooth gradient, and is suitable for tasks that require predicting probabilities as outputs. Its limitation is that it stops the DNN from learning and makes the DNN suffer from the vanishing gradient problem as the gradient value approaches zero.

The tanh activation function also has an S-shape like the sigmoid function, but with the difference in output range of −1 to 1. That is, with tanh, the larger the input, the closer the output value is to 1. This function is widely used for hidden layers of a DNN, because it can help to center the data and make the learning for the next layer easier. However, it also faces the same problem of vanishing gradients as the sigmoid function. However, in practice, the tanh activation function is more preferred than sigmoid due to its zero-centered nature.

The ReLU activation function, which stands for Rectified Linear Unit, is another most important and popular activation function. Its main feature is that it does not activate all the nodes at the same time, and only the nodes with an output larger than 0 will be activated. Therefore, this function is computationally efficient, compared to the sigmoid and tanh activation functions. In addition, it facilitates the convergence of gradient descent towards the global minimum of the loss function. Its limitation is that it may cause possible dead nodes due to the negative side of the curve making the gradient value zero.

Therefore, the leaky ReLU activation function, which is an improvement of ReLU, has been proposed to solve the dying ReLU problem. It has a small positive slope for the negative side, which enables back-propagation for negative inputs. This way, the gradient of the negative side of the curve will be a nonzero value, and the problem of dead nodes is solved. The limitation of this activation function is that it makes the learning of the DNN time-consuming.

#### 2.2.3. Stochastic Gradient Descent (SGD)

The SGD is an efficient approach for fitting linear classifiers or regressors under convex loss functions, especially in high-dimensional optimization. Therefore, it has been widely and successfully used as an important optimization method for training a DNN [7,14,15,16]. It has the advantages of high efficiency and ease of implementation, but also the disadvantages of requiring some hyperparameters such as the regularization parameter and the number of iterations, and being sensitive to feature scaling.

#### 2.2.4. Back-Propagation (BP)

The BP, which is short for “backward propagation of errors”, is the most prominent algorithm to train a DNN. Strictly, it refers only to the algorithm for computing the gradient, not how to use the gradient. However, loosely and generally, it refers to using the mean squared error and the SGD to fine-tune the weights of a DNN. Specifically, it calculates the gradient of a loss function with respect to all the weights in the DNN by the chain rule, and employs the SGD to decide how to use the gradient to properly tune the weights. A DNN’s weights are iteratively tuned until the desired output is achieved.

### 2.3. Learning Scenarios of Deep Learning

#### 2.3.1. Supervised Learning

Supervised learning is a learning paradigm that uses a set of labeled examples as training data and makes predictions for all unseen points [17]. Supervised algorithms are expected to learn the mapping between pairs of inputs and output values, also called annotations or labels. This scenario includes two types of problem: classification and regression.

#### 2.3.2. Semi-Supervised Learning

Semi-Supervised learning (SSL) aims to learn predictive models that make use of both labeled and unlabeled data. SSL provides a feasible solution in the setting where unlabeled data are easily accessible, but labels are difficult to obtain [18]. By exploring the pattern in additional unlabeled data, SSL methods can improve the learning performance. Deep SSL has dominated this research area in recent years [19,20,21].

#### 2.3.3. Unsupervised Learning

In contrast to supervised learning, unsupervised learning constructs models where only unlabeled data are available [22]. The key of unsupervised methods is to discover hidden patterns and discriminative feature representations without human intervention. Clustering and dimensionality reduction are examples of unsupervised learning problems.

#### 2.3.4. Reinforcement Learning

Different from supervised learning, reinforcement learning refers to the learning scenario where the learner receives rewards after a course of actions by interacting with the environment, and then determines the optimal actions by maximizing the rewards to achieve the goal [17]. With different states of the environment, the problem can be divided into two settings: The planning problem and learning problem.

### 2.4. Training Strategy and Performance

#### 2.4.1. Learning Rate

Learning rate is one of the most important hyperparameters when configuring a neural network. It controls how much a model is changed based on the estimated error each time the model weights are updated [23]. Choosing an appropriate learning rate is very challenging, because a very small value may cause the training process to be too long and get stuck, while a very large value may result in learning a suboptimal set of weights or with an unstable training process. A typical solution to choose the appropriate learning rate is to reduce the learning rate during training. Currently, there are three kinds of popular ways to achieve this: constant, factored, and exponential decay.

#### 2.4.2. Weight Decay

Weight decay is a regularization technique applied to the weights of a neural network for shrinking the weights during back-propagation. It works by adding a penalty term, which is usually the L2 norm of the weights, to the loss function. It can help to prevent overfitting and avoid exploding gradient.

#### 2.4.3. Dropout

Dropout is widely used to prevent overfitting by randomly dropping out neural units in a neural network. It is a strong regularization to prevent complex co-adaptations on training data [24]. More technically, at each training stage, individual nodes are either dropped out of the network with probability 1−p or kept with probability *p*, leading to a reduced network. Dropout forces a neural network to learn more robust features and roughly doubles the number of iterations required to converge, but the training time for each epoch is less.

#### 2.4.4. Early Stopping

Early stopping is a training strategy used to reduce overfitting without compromising on model accuracy. The underlying idea behind early stopping is to stop training before a model starts to overfit. There are mainly three strategies for early stopping: training models on a preset number of epochs, stop when the loss function update becomes very small, and observing the changes of training and validation errors with the number of epochs.

#### 2.4.5. Batch Normalization

Batch normalization is a technique to standardize the inputs to a neural network for stabilizing the learning process and reducing the number of training epochs required to train deep networks [25]. With batch normalization, a network can use higher learning rates, achieve better results, and the training can be faster. It also makes activation functions viable by regulating the inputs to them, and adds noise which reduces overfitting with a regularization effect.

#### 2.4.6. Data Augmentation

Data augmentation refers to a set of techniques to artificially increase the amount of training data by generating new data from existing data. It is a low-cost and effective method to improve the performance and accuracy of deep learning models in data-constrained environments. For visual data, alterations such as cropping, rotating, scaling, flipping, contrast changing, adding noise are effective and popular data augmentation methods. For other kinds of data, data augmentation is not as popular as for visual data, due to the complexity of the data. Some advanced models such as GANs are popular for data augmentation [26,27,28].

### 2.5. Deep Learning Platforms and Resources

#### 2.5.1. Deep Learning Platforms

The two currently most renowned end-to-end open source platforms for deep learning are TensorFlow [29] and PyTorch [30]. They provide comprehensive and flexible ecosystems of tools, libraries, and community resources that let engineers and researchers easily build and deploy deep learning powered applications.

TensorFlow (https://www.tensorflow.org/ accessed on 2 November 2022) is developed by researchers and engineers at Google and was released in 2015. It is a symbolic math library and is best suited for data flow programming across a wide variety of tasks. It provides multiple abstraction levels for building and training a DNN. In addition, it has adopted Keras (https://keras.io/ accessed on 2 November 2022), which is a functional API that extends TensorFlow and allows users to easily code some high-level functional sections. It provides system-specific functionality such as pipelining, estimators, and eager execution, and supports various topologies with different combinations of inputs, output, and layers.

PyTorch (https://pytorch.org/ accessed on 2 November 2022) is based on Torch and is relatively new compared to TensorFlow. It is developed by researchers at Facebook and was released in 2017. It is well known for its simplicity, ease of use, flexibility, efficient memory usage, and dynamic computational graphs. Due to its computation power and native programming feeling, PyTorch is emerging as a winner. Furthermore, it has a large community of developers and researchers who have built rich and powerful tools and libraries to extend PyTorch. Some popular libraries include GPyTorch, BoTorch, and Allen NLP.

Other frameworks include Caffe [31], Torch [32], DL4j (https://deeplearning4j.konduit.ai/ accessed on 2 November 2022, Neon (https://github.com/NervanaSystems/neon accessed on 2 November 2022, Theano [33], MXNet [34], and CNTK [35]. The choice of which platform is superior has always been controversial, but PyTorch and TensorFlow are undoubtedly the two most popular deep learning frameworks today.

#### 2.5.2. Codes and Pretrained Models

While TensorFlow and PyTorch have provided official tutorials on how to use them, topic-specific tutorials for different levels are beneficial and complementary. There are many reputable courses online, for example, Practical Deep Learning for Coders (https://course.fast.ai/ accessed on 2 November 2022), which provides practical programming skills and an easy-to-use code library for most important deep learning techniques. Furthermore, it is free and without ads, and is designed for learners with various background levels. More useful courses can be found at the collection of AI Curriculum from top universities (https://github.com/Machine-Learning-Tokyo/AI_Curriculum accessed on 2 November 2022). A comprehensive collection of deep learning books, videos, lectures, workshops, datasets, tools, etc., is available on GitHub (https://github.com/ChristosChristofidis/awesome-deep-learning accessed on 2 November 2022).

Open source code can greatly help to learn deep learning and improve the efficiency of the learning. The distinguished Papers With Code website https://paperswithcode.com/ (accessed on 2 November 2022) collects new research papers and their corresponding open source codes, as well as the latest trending directions and state-of-the-art results across many standard benchmarks.

As we will describe in later sections, utilizing pretrained models is an important technique in transfer leaning and can greatly improve the efficiency of deep leaning. A collection of pretrained models is available for both TensorFlow (https://github.com/tensorflow/models accessed on 2 November 2022) and Pytorch (https://pytorch.org/vision/stable/models.html accessed on 2 November 2022). The AI community Hugging Face (https://huggingface.co/accessed on 2 November 2022) also provides a huge collection of pretrained models as well as the codes to train these models. The website Model Zoo (https://modelzoo.co/ accessed on 2 November 2022) is also a great place to discover pretrained models and open source deep learning codes.

#### 2.5.3. Computing Resources

Training deep learning models requires relatively high computing resources. Therefore, open source web-based development environments that run entirely in the cloud are very helpful for average researchers. The two currently popular web applications for interactive computing are Jupyter Notebook (https://jupyter.org/ accessed on 2 November 2022) and Colab (https://colab.research.google.com/ accessed on 2 November 2022). They are very similar, and both require zero configuration, provide access to GPUs free of charge, and support most popular machine learning libraries. They are easy to use and to create documents that contain live code, equations, visualizations, and text. Furthermore, their flexible interfaces allow users easily to configure, arrange, and share workflows for team work.

Tracking and visualizing metrics such as loss and accuracy during the model training is a vital process of training a DNN. A predominant toolkit for this purpose is Tensorboard (https://www.tensorflow.org/tensorboard accessed on 2 November 2022), which works for both TensorFlow and Pytorch. In addition to the above functions, it can visualize model graphs, views histograms of weights, biases, or other tensors as they change over time, project embeddings to a lower dimensional space, display images, text, and audio, and so on.

## 3. Deep Learning Models and Methods

### 3.1. Convolutional Neural Network (CNN)

#### 3.1.1. Introduction of CNN

The design of convolutional networks was inspired by biological processes where the pattern of connections between neurons resembles the organization of the human visual cortex: individual cortical neurons respond only to stimuli in the receptive fields, which partially overlap to cover the entire field of view [36].

**Figure 3:**
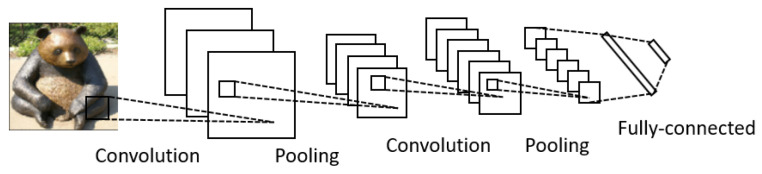
A typical CNN architecture.

A typical CNN consists of several convolutional layers and pooling layers followed by fully connected layers at the end (Figure 3). The input of a CNN is a tensor arranged in four dimensions (N×h×w×c), where *N* denotes the number of inputs, *h* and *w* are the height and width of the input, and *c* the depth or number of channels of the input (c=3 for an RGB image). The convolutional layer convolves the input with *k* kernels/filters of size ($k_{h}\times k_{w}\times k_{c}$), where $k_{h}<h$, $k_{w}<w$, and $k_{c}\leq c$, and generates and passes *k* feature maps to the following layer. These kernels share the same parameters and form the base of local connections. The convolution operation performs a dot product (usually the Frobenius inner product) of the kernel with a small region of the layer’s input matrix each time, then an activation function (usually the ReLU function) is applied. As the kernel slides along the input matrix, a feature map is generated. The pooling layers reduce the dimension of the feature maps by subsampling, thus decreasing the number of parameters for training. The pooling operation usually takes the maximum (max pooling) or average value (average pooling) of the local cluster of neurons (local pooling) or all neurons (global pooling) in the feature map. The last few layers of a CNN are fully connected layers, as in a multilayer perception that connect every neuron in one layer to every neuron in the following layer. Through these layers, the CNN extracts high-level representations from the input data, and its final layer outputs the probabilities that the instance belongs to each class.

CNNs improve the fully connected networks in three major aspects: (1) local connections, (2) weight sharing, and (3) subsampling. These mechanisms significantly reduce the number of parameters, speed up convergence, and make CNN an outstanding algorithm in the field of deep learning. CNNs are particularly popular in computer vision applications since they fully exploit the two-dimensional structure of the input image data [37].

Since its first introduction, the CNN design has received widespread attention from researchers, and various variant models and improvements have been proposed. Next, we introduce several representative CNN models and their main contributions. Table 2 summarizes these models and following works.

#### 3.1.2. AlexNet

AlexNet [37] consists of eight layers: five convolutional layers, some of which followed by max-pooling layers, concatenated with three fully connected layers. It uses the ReLU activation function, which shows improved training performance over tanh and sigmoid which are prone to the vanishing gradient problem [53] (e.g., the derivative of sigmoid becomes very small in the saturating region, and therefore, the updates to the weights almost vanish). A dropout layer is used after every fully connected layer, reducing overfitting. AlexNet was one of the first deep neural networks to push ImageNet classification accuracy up by a significant amount (a top five accuracy of 80.2%) in comparison to traditional methods. The depth of the model was essential for its high performance, and while computationally expensive, training was made feasible by the utilization of GPUs.

#### 3.1.3. VGG

VGG [39] improves over AlexNet by replacing large size kernels (11 and 5 in the first and second convolutional layer, respectively) with multiple 3×3 kernels one after another. The idea behind this is that with a given receptive field, stacking multiple kernels of smaller size is better than using one kernel of larger size. This is because multiple nonlinear layers increase the depth of the network, which enables it to learn more complex features at a lower cost. In addition, the 3×3 kernels help retain finer representations of the input. In VGG-D, blocks with the same kernel size are applied multiple times to extract more complex and representative features. This concept of blocks or modules became a common theme in the networks after VGG. It achieved top five accuracy of 91.2% on ImageNet.

#### 3.1.4. GoogLeNet (Inception)

**Figure 4:**
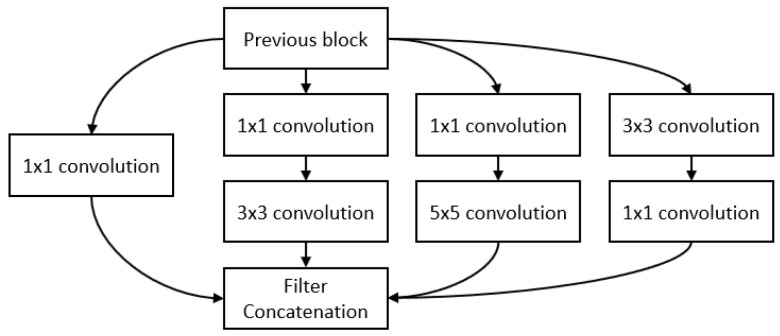
The inception module in GoogLeNet.

GoogLeNet [38] introduces the inception module to form a sparse architecture rather than the previous dense connection architecture to reduce the computation requirement of training deep networks such as VGG. It builds on the idea that most of the activations in a deep network are either unnecessary or redundant because of correlations between them. Therefore, the most efficient architecture of a deep network will have a sparse connection between the activations, rather than a dense connection architecture. Thus, the inception module (Figure 4) approximates a sparse CNN with a normal dense construction. Since only a few neurons are effective, the width and number of the convolutional filters of a particular kernel size is kept small. Convolutions of different sizes are used to capture features at varied scales (5×5, 3×3, 1×1). A bottleneck layer (1×1 convolutions) is introduced for massive reduction of the computational cost. All these changes allow the network to have a large width and depth. GoogLeNet is built on top of the inception blocks and it replaces the fully-connected layers at the end with a simple global average pooling which averages out the channel values across the 2D feature map. This drastically reduces the total number of parameters. It achieves 93.3% top five accuracy on ImageNet and is much faster to train than VGG.

#### 3.1.5. ResNet

**Figure 5:**
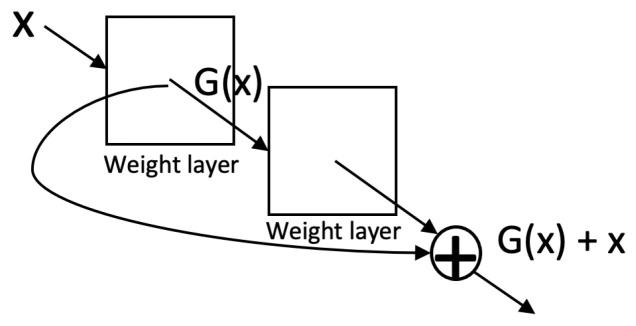
Illustration of a residual learning module.

ResNet [41] was proposed to solve the vanishing gradient problem [54] and degradation problem. The vanishing gradient prevents the update of the weights and hinders convergence from the beginning due to the increased depth. The degradation problem refers to the phenomenon that as the network depth increases, accuracy gets saturated and then degrades rapidly (this is not caused by overfitting but adding more layers leads to higher training error) [41]. Degradation of training accuracy indicates that not all systems are similarly easy to optimize. Hence, the residual learning framework is designed to recast the original mapping H(x) into a residual mapping which is easier to optimize than the original mapping. The residual module (Figure 5) creates a shortcut connection between the input and output to the module, implying an identity mapping, thus allowing the stacked nonlinear layers to fit a residual mapping G(x):=H(x)−x. With these shortcuts, the residual module helps to build deeper neural networks as large as a network depth of 152. In addition, ResNet adopts a global average pooling followed by the classification layer as in GoogLeNet. It achieves better accuracy (95.51% top five accuracy with ResNet-152) than VGGNet and GoogLeNet while being computationally more efficient than VGGNet.

#### 3.1.6. DenseNet

**Figure 6:**
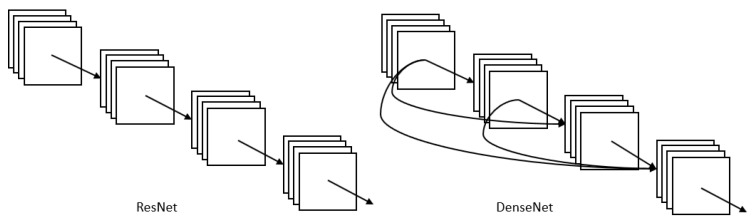
DenseNet block vs. ResNet block.

DenseNet [42] is one of the new discoveries in neural networks for visual object recognition. DenseNet is quite similar to ResNet but with some fundamental differences: ResNet uses an additive method to merge the previous layer (identity) with the future layer, whereas DenseNet concatenates the output of the previous layer with the future layer. For ResNet, the identity shortcut that stabilizes training also limits its representation capacity, while DenseNet has a higher capacity with multilayer feature concatenation. In DenseNet, each layer obtains additional inputs from all preceding layers and passes on its own feature maps to all subsequent layers (Figure 6). With concatenation, each layer is receiving collective knowledge from all preceding layers. However, the dense concatenation requires higher GPU memory and more training time.

#### 3.1.7. UNet

**Figure 7:**
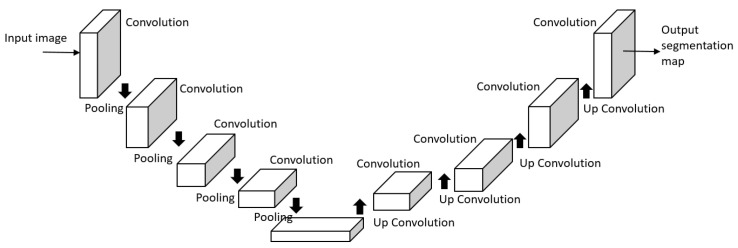
UNet architecture.

UNet [43] is an architecture originally developed for biomedical image segmentation and is now one of the most popular approaches in semantic segmentation tasks. UNet is a U-shaped encoder-decoder network architecture consisting of four encoder blocks and four decoder blocks that are connected via a bridge (Figure 7). The encoder network (contracting path) acts as the feature extractor and learns an abstract representation of the input image through a sequence of the encoder blocks. It halves the spatial dimensions and doubles the number of filters at each encoder block. The decoder network takes the abstract representation and generates a semantic segmentation mask. It doubles the spatial dimensions and half the number of feature channels.

#### 3.1.8. Mask R-CNN

**Figure 8:**
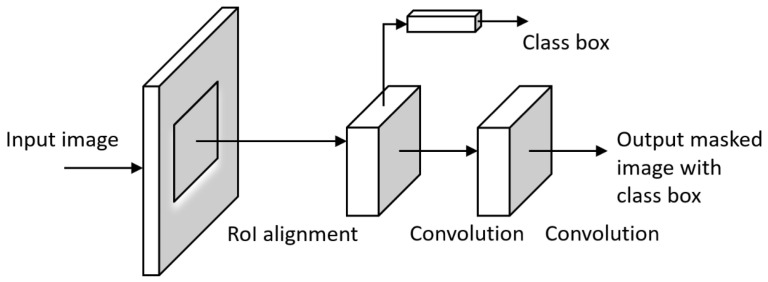
Mask R-CNN model.

Mask Region-based CNN (mask R-CNN) [47] is the state-of-the-art in terms of image segmentation. It detects objects in an image and generates a high-quality segmentation mask for each instance. Mask R-CNN can deal with two types of image segmentation: semantic segmentation separates the subjects of the image from the background without differentiating object instances; and instance segmentation accentuates the subjects by detecting all objects in the image while segmenting each instance. The R-CNN is a type of model that utilizes bounding boxes across the object regions and then evaluates CNNs independently on all the Regions of Interest (RoI) to classify multiple image regions into the proposed classes. An improved version of R-CNN is Fast R-CNN [55] which extracts features using RoI Pooling from each candidate box and performs classification and bounding-box regression. Faster R-CNN [44] was then designed to add the attention mechanism with a region proposal network to the Fast R-CNN architecture. Mask R-CNN is an extension of Faster R-CNN by adding a branch for predicting an object mask in parallel with the existing branch for bounding box recognition (Figure 8). It outputs a class label, a bounding-box offset, and the object mask, where the mask output requires the extraction of a fine spatial layout of an object. The key element of Mask R-CNN is the pixel-to-pixel alignment, which is the main missing piece of Fast/Faster R-CNN. Mask R-CNN is simple to implement and train given the Faster R-CNN framework, which facilitates a wide range of flexible architecture designs. Additionally, the mask branch only adds a small computational overhead, enabling a fast system and rapid experimentation.

#### 3.1.9. YOLO

YOLO [46] is a popular model for real-time object detection, which concerns what and where objects are inside a given image. The algorithm applies a single neural network to the full image, and then divides the image into regions and predicts bounding boxes and probabilities for each region. These bounding boxes are weighted by the predicted probabilities. YOLO is popular because it achieves high accuracy while also being able to run in real-time. The algorithm ‘only looks once’ at the image in the sense that it requires only one forward propagation pass through the neural network to make predictions. After non-max suppression (which makes sure the object detection algorithm only detects each object once), it then outputs recognized objects together with the bounding boxes. With YOLO, a single CNN simultaneously predicts multiple bounding boxes and class probabilities for those boxes. It trains on full images and directly optimizes detection performance.

### 3.2. Recurrent Neural Network (RNN)

#### 3.2.1. Introduction of RNN

The RNN is a type of artificial neural network that is especially suitable for processing sequential information such as natural languages or time series data such as videos [56,57]. Applications of RNNs include handwriting recognition [58], speech recognition [59], gesture recognition [60], image captioning [61], natural language processing [62] and understanding [63], sound event prediction [64], tracking and monitoring [65,66,67,68,69], etc.

**Figure 9:**
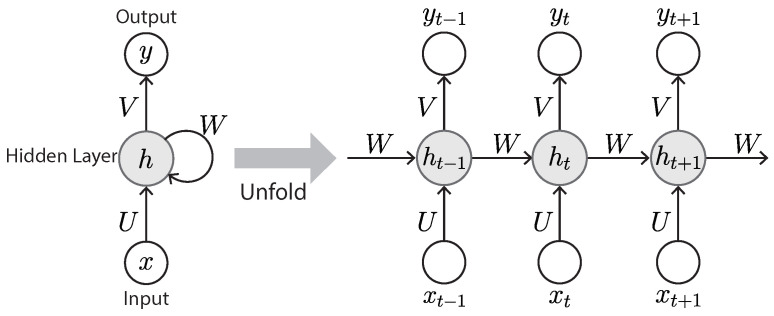
Diagram of an RNN. x,y represent the input and output, respectively, *t* is the time, *h* is the memory unit of the network, and U,V,and W are weight matrices.

Unlike traditional neural networks, the RNN can exploit sequential information by means of a connection that acts as feedback to prior layers (Figure 9). The most distinguished characteristic of an RNN is that it has memory, taking information from prior inputs to influence the current input and output. Because of this unique characteristic, an RNN can remember important information of the input, which allows it to predict with great precision what will happen next. That is why the RNN is the method of choice for processing sequential data. Another salient characteristic of the RNN is that it shares the same weight parameters within each layer of the network, whereas a normal feed-forward network has different weights on each node.

The RNN employs the back-propagation through time (BPTT) algorithm to adjust and fit the parameters of the model [70,71,72]. BPTT is almost the same as the standard BP, except that it sums errors at each time step, while BP does not need to sum errors because it does not share parameters between each layer. This also makes the RNN have two main issues of vanishing gradients and exploding gradients [73]. In other words, gradients may decay or explode exponentially due to the multiplications of a large number of small or large gradients during training over time. Therefore, the RNN tends to forget the previous inputs as the new inputs come in. One solution to these issues is to clip the gradient and scale the gradient. Long Short-Term Memory (LSTM) [63] (see below) is proposed to handle this issue by providing memory blocks in its recurrent connections.

#### 3.2.2. Bidirectional Recurrent Neural Network (BRNN)

**Figure 10:**
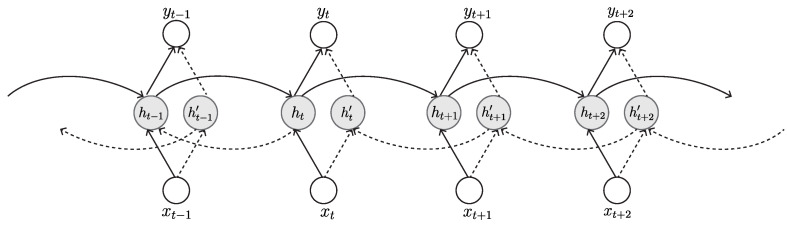
Diagram of a BRNN. x,y represent the input and output, respectively, $h,h^{\prime }$ represent the two bidirectional memory units, and *t* is the time. Solid arrows represent data forward propagation, and dashed arrows represent data back propagation.

The BRNN was firstly invented in 1997 by Schuster and Paliwal for increasing the amount of input information available to the network [74]. It is a variant architecture of the RNN. While the classical RNN can learn only from previous layers to predict the current state, the BRNN learns from future data to improve its accuracy. This is achieved by a structure of connecting two hidden layers of opposite direction to the same output (Figure 10). BRNNs are especially beneficial in cases where the context of the input is required. For example, in handwriting recognition, performance can be improved by knowing the letters before and after the current letter [75]. The BRNN is more common in supervised learning rather than semi-supervised or unsupervised learning because it is difficult to compute a reliable probabilistic model.

The training of a BRNN is similar to the BPTT algorithm. However, since there are forward and backward passes, simultaneously updating the weights for the two processes leads to erroneous results. Therefore, to update forward and backward passes separately, the forward and backward states are firstly processed in the forward pass, and then the output values are passed. Subsequently, the reverse takes place for the backward pass; that is, the output values are processed first, and then the forward and backward states are processed. Finally, the weights are updated after the completion of both forward and backward passes.

#### 3.2.3. Long Short-Term Memory (LSTM)

**Figure 11:**
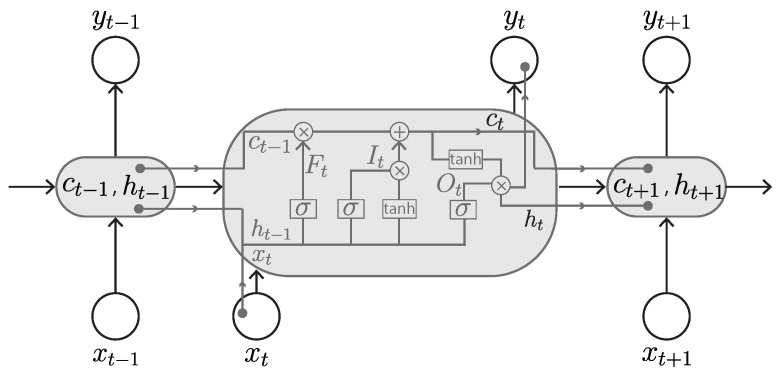
Diagram of a LSTM memory cell. c,h are the cell state and hidden state, respectively, *t* is the time, and $F_{t},I_{t},O_{t}$ are the forget gate, input gate, and output gate, respectively.

The LSTM was proposed by Hochreiter and Schmidhuber, and has been widely used for many applications [76]. It is an improved version of RNN, with the memory blocks (also called cells) able to let new information in, forget information, and give information enough importance to affect the output. It uses a mechanism of ‘gates’ for controlling its memory process (Figure 11). There are three gates: input gate, output gate, and forget gate. The input gate is responsible for accepting new information and information from the previous hidden state. The forget gate is responsible for deciding the storage or removal of information based on the learned weights. The output gate is responsible for determining the value of the next hidden state. This gate mechanism regulates the flow of information in the RNN and resolves the short-term memory issue, thus enabling an RNN to hold its value for a sufficient amount of time.

The gates in the LSTM are modeled in the form of sigmoid function. To decide which information can pass through and what information can be discarded, the short-term memory and input pass through the sigmoid function, which transforms the values to be between 0 and 1, where 0 indicates the information is unimportant and 1 indicates the information is valuable. The use of the sigmoid function also guarantees that the gates can be back-propagated. The LSTM keeps the gradients steep enough and thus solves the issue of vanishing gradients in RNNs. This also makes its training comparatively short and its accuracy comparatively high.

#### 3.2.4. Gated Recurrent Unit (GRU)

**Figure 12:**
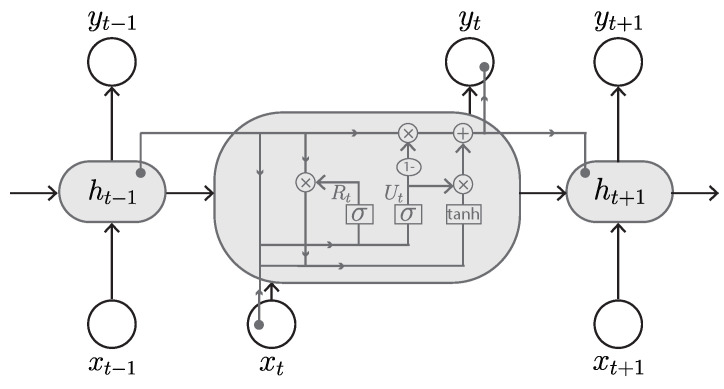
Diagram of a GRU memory cell. x and y are the input and output, respectively, *h* is the hidden state, *t* is the time, and $R_{t}\;and\;U_{t}$ are the reset gate and update gate.

The GRU, proposed by Cho et al. in 2014 [56], is also a variant of RNN and is very similar to the LSTM and, in some cases, produces equally good results [77]. It has two gates, an update gate and a reset gate (Figure 12), rather than three gates as in LSTM. The reset gate is responsible for the short-term memory and controls what information goes out or is discarded. The update gate is responsible for long-term memory and regulates information to be retained from previous memory as well as the new memory to be added. In addition, the GRU uses hidden states rather than separate cell states in LSTM to regulate the flow of information. Therefore, due to the reduced number of parameters and its simpler architecture, GRU is faster to train with high effectiveness and accuracy. The GRU is also able to address the short-term memory problem of RNN and to effectively hold long-term dependencies in sequential data.

#### 3.2.5. RNN with Attention

Introducing attention to RNNs is probably the most significant innovation in sequential models in recent times. Attention refers to the ability of a model to focus on specific elements in the data. As mentioned, RNNs try to remember the entire input sequence through a hidden unit before predicting the output. However, compressing all information into one hidden unit may lead to information loss, especially for long sequences. To help the RNN focus on the most important elements of the input sequence, the attention mechanism assigns different attention weights to each input element. These attention weights designate how important or relevant a given input sequence element is at a given time step.

The first attention mechanism developed for RNNs was proposed by Bahdanau et al. [78] in 2014, who used it for language translation. Later, several RNN variants with attention mechanism were proposed. Examples include the dual state attention based RNN for time series prediction [79], the attention based GRU for visual question answering [80], and the outstanding attention-LSTM for Google’s neural machine translation system [81]. The success of attention-LSTM has inspired more research of neural networks based on attention mechanism, and with more and more powerful computing resources becoming available, state-of-the-art models now typically use a memory-hungry architectural style called transformers (Section 3.7).

### 3.3. AutoEncoder (AE)

#### 3.3.1. Introduction of AE

The AE is a type of artificial neural network that can learn data representation in an unsupervised manner [13]. It is a specific type of feed-forward neural network where the input is the same as the output. Its aim is to learn a low-dimensional representation (also called latent-space representation or encoding) of high-dimensional data by training the network to capture the most important elements of the inputs, usually for dimensionality reduction. By using it as an encoding and decoding technique, and combing it with other DNNs such as CNN and RNN, the AE concept has been extensively applied for data (images, audio, etc.) denoising [82,83], information retrieval [84,85], image inpainting and enhancement [86,87], and anomaly detection [88,89].

**Figure 13:**
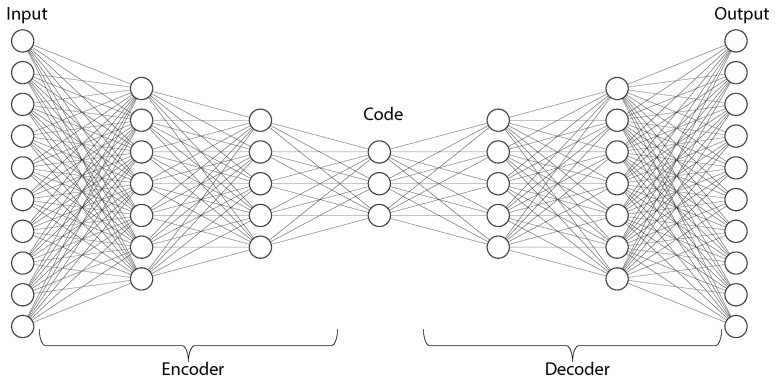
Diagram of an AE.

A classical AE consists of three components named encoder, code, and decoder (Figure 13). The encoder maps the input data to the feature space and produces the code, while the decoder then reconstructs the data by mapping this code back to the data space. The encoder is essentially a fully-connected neural network (though other types of networks such as CNNs can also be used), and the decoder has a similar mirror network structure as the encoder. The code is a compressed representation of the input and is important to prevent the AE from memorizing the input and overfitting on the data.

Since the goal of an AE is to get an output identical to the input, it can be trained by minimizing a reconstruction loss formulated as:

(1) $$L_{A}\left(x,\hat{x}\right)=||x-\hat{x}{\left|\right|}^{2},$$

where *x* is the input and $\hat{x}$ is the corresponding reconstruction by the AE. It is trained the same way as a DNN via BP, and also has the vanishing gradient problem because gradients may become too small as they go back through many layers of the AE.

#### 3.3.2. Sparse AE (SAE)

The SAE is a regularized AE proposed by Ranzato et al. [90] to learn sparse representations. It is used to learn latent representations instead of redundant information of the input data, and has been shown to improve performance on classification tasks. A SAE selectively activates regions of the network, depending on the input data. As a result, it is restrained to memorize the input data but can effectively extract features from the data. More specifically, a SAE adds a nonlinear sparsity between its encoder and decoder to force the code vector into a quasi-binary sparse code. There are two ways to impose this sparsity regularization, and both are adding a constraint term to the loss function. By adding an L1 regularization as the constraint term, the loss function is formulated as:

(2) $$L_{S}\left(x,\hat{x}\right)=L\left(x,\hat{x}\right)+\alpha \sum \left|a^{h}\right|,$$

where $L(x,\hat{x})$ is computed using Equation (1), α is the parameter to control the regularization strength, and *a* is the activation of the hidden layer *h*. By adding a KL-divergence as the constraint term, the loss function is formulated as:

(3) $$L_{S}\left(x,\hat{x}\right)=L\left(x,\hat{x}\right)+\beta \mathrm{KL}(\rho ||\hat{\rho }),$$

where $L(x,\hat{x})$ is computed using Equation (1), β is the parameter to control the regularization strength, $\hat{\rho }$ is the average activation of the code over the input data, ρ is a sparsity hyperparameter, and $\mathrm{KL}(\rho ||\hat{\rho })$ is the KL divergence of $\left(\rho ||\hat{\rho }\right)$, with minimum at $\hat{\rho }=\rho$.

#### 3.3.3. Contractive AE (CAE)

The CAE is another variant of the classical AE, which adds a contractive regularization to the code to improve its feature representation capability [91]. Its basic principle is that similar inputs should have similar encodings and similar latent space representations. To this end, CAE requires the derivative of the hidden layer activations to be small with respect to the input. Thus, the mapping from the input to the representation will converge with higher probability. The loss function of the CAE is defined as:

(4) $$L_{C}\left(x,\hat{x}\right)=L\left(x,\hat{x}\right)+\gamma ||J\left(x\right){\left|\right|}_{F}^{2},$$

where $L(x,\hat{x})$ is computed using Equation (1), γ is the parameter to control the regularization strength, J(x) represents the Jacobian matrix of the encoder, and ${\left||J\left(x\right)|\right|}_{F}^{2}$ is the square of the Frobenius norm of the Jacobian matrix. It is worth mentioning that these two terms in the CAE loss function contradict each other. While the reconstruction loss $L(x,\hat{x})$ aims to distinguish the difference between two inputs and observe changes in the data, the regularization ${\left||J\left(x\right)|\right|}_{F}^{2}$ aims to allow the model to ignore changes in the input data. However, a loss function with these two terms enables the hidden layers of the CAE to capture only the most essential information.

#### 3.3.4. Denoising AE (DAE)

The DAE was originally proposed by Vincent et al. [92,93] based on the AE for removing noise of the input. Now, it has become an important and essential tool for feature extraction and selection. Different from the above types of AEs, the DAE does not have the input image as its ground truth. Its basic idea is to slightly corrupt the input data but still use the uncorrupted data as target output. This way, it can force the DAE to recover a noise-free version of the input data. Furthermore, a DAE model cannot simply learn a map that memorizes the input and overfits the data because the input and target output are no longer the same. Essentially, a DAE gets rid of noise with the help of nonlinear dimensionality reduction. The loss function used by the DAE is expressed as:

(5) $$L_{D}\left(x,\hat{x^{\prime }}\right)=||x-\hat{x^{\prime }}{\left|\right|}^{2},$$

where $x^{\prime }$ is the corrupted version of input *x*, and $\hat{x^{\prime }}$ is the reconstruction by the DAE. A DAE can exploit the statistical dependencies inherent in the input data and remove the detrimental effects of noisy inputs.

#### 3.3.5. Variational AE (VAE)

While AEs can learn a representative code from the input data and reconstruct the data from this compressed code, the distribution of this compressed code remains unknown and cannot be expressed in a probabilistic fashion. The VAE [94] is designed to handle this issue and learn to format the code as a probability distribution. This way, the learned code can be easily sampled and interpolated to generate new unseen data. Therefore, the VAE is a kind of deep generative model. The VAE makes the code to be a Gaussian distribution, so that the encoder can be trained to return its mean μ and variance $\sigma^{2}$. The loss function for VAE training is defined as:

(6) $$L_{V}\left(x,\hat{x}\right)=L\left(x,\hat{x}\right)+\mathrm{KL}\left(N\left(\mu ,\sigma \right),N\left(0,1\right)\right),$$

where $L(x,\hat{x})$ is computed using Equation (1), KL(N(μ,σ),N(0,1)) is a regularization term on the learned code to force the distribution of the extracted code to be close to a standard normal distribution. The reason why an input is encoded as a distribution with some variance rather than a single point is that it expresses the latent space regularization very naturally. Sampling from this latent distribution and feeding it to the decoder can lead to new data being generated by the VAE.

### 3.4. Restricted Boltzmann Machine (RBM)

The RBM was invented by Hinton in 2007 for learning a probability distribution over its set of inputs [95]. It is a generative stochastic artificial neural network that has wide applications in different areas such as dimensionality reduction [96], classification [97], regression [98], collaborative filtering [99], feature learning [100], and topic modeling [101].

**Figure 14:**
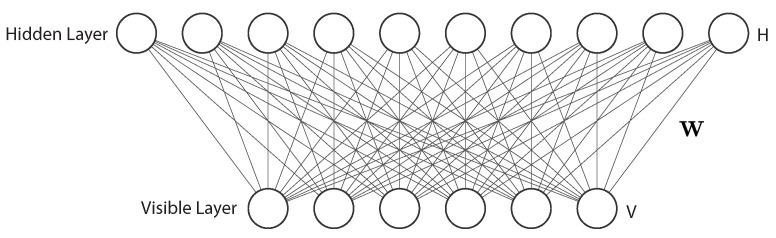
Diagram of an RBM. V,H, and W represent the state vector of the visible layer, the state vector of the hidden layer, and the weight matrix between hidden and visible layers, respectively.

A classical RBM has two layers, named visible layer and hidden layer (Figure 14). The visible layer has input nodes to receive input data, while the hidden layer is formed by nodes that extract feature information from the data and output a weighted sum of the input data [102]. An important and unique characteristic of the RBM is that the output generated by the hidden layer is further processed to become a new input to the visible layer. This process is called reconstruction or backward pass, and is repeated until the regenerated input is aligned with the original input. This way, an RBM is able to learn a probability distribution over the input. In an RBM, there is no typical output layer. In addition, every node can be connected to every other node, and there are no connections from visible to visible or hidden to hidden nodes.

An RBM is also a generative model. It represents a probability distribution by the connection weights learned from the data. Denote the *m* visible nodes as $V=(v_{1},v_{2},\cdots ,v_{m})$ and *n* hidden nodes as $H=(h_{1},h_{2},\cdots ,h_{n})$. In a binary RBM, the random variables (V,H) take values $\left(v,h\right)\in {\left\{0,1\right\}}^{m+n}$, and the joint probability distribution is given by the Gibbs distribution $p\left(v,h\right)=1/Ze^{-E(v,h)}$ with the energy function defined as [103]:

(7) $$E\left(v,h\right)=-\sum_{i=1}^{n}\sum_{j=1}^{m}w_{ij}h_{i}v_{j}-\sum_{i=1}^{n}b_{i}h_{i}-\sum_{j=1}^{m}c_{j}v_{j},$$

where $Z=\sum_{v,h}e^{-E(v,h)}$ is the normalization factor, i∈1,2,⋯,n and j∈1,2,⋯,m, $w_{ij}$ is a weight associated with the edge between nodes $v_{j}$ and $h_{i}$, and $b_{i}$ and $c_{j}$ are biases associated with the *i*th visible and the *j*th hidden variable, respectively. The RBM has proven to be capable of achieving highly expressive marginal distributions [104].

### 3.5. Generative Adversarial Network (GAN)

#### 3.5.1. Introduction of GAN

The GAN was firstly proposed by Goodfellow et al. [105] and has become one of the most popular generative adversarial models. Its purpose is to learn the distribution of input data and thus enable the network to generate new data from that same distribution. Since the GAN was proposed, it has gained much attention in various areas such as synthetic training data [106], image and audio style transfer [107], music generation [108], text to image generation [109], super-resolution [110], semantic segmentation [111], natural language processing [112], and predicting the next frame in a video [113].

**Figure 15:**
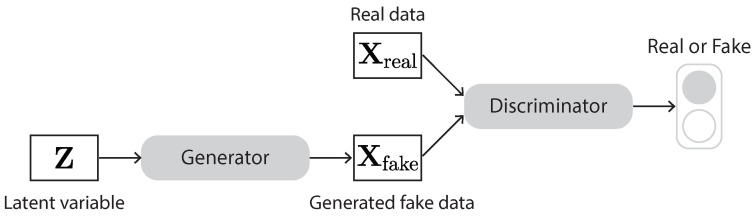
Diagram of a GAN.

A GAN is basically composed of two neural networks, named generator and discriminator (Figure 15). The generator takes a random vector sampled from a noise distribution as input and generates samples. The discriminator takes the generated samples and real samples as input and tries to distinguish them as real or fake. These two networks compete with each other. The goal of the generator is to generate fake samples that are hard for the discriminator to distinguish from real samples. The goal of the discriminator is to beat the generator by identifying whether its received samples are fake or real. This competition between the generator and discriminator goes on until the generator manages to generate fake samples that the discriminator cannot distinguish from real ones.

This zero-sum game is modeled as an optimization problem by:

(8) $$\min_{G}\;\max_{D}\;L\left(D,G\right),$$

where *D* and *G* denote the generator and discriminator, respectively, and

(9) $$L\left(D,G\right)=E_{x∼p_{\mathrm{data}}\left(x\right)}\left[\log\left(D\left(x\right)\right)\right]-E_{x∼p_{z}\left(z\right)}\left[1-\log\left(D\left(G\left(z\right)\right)\right)\right],$$

where *x* is the input data, $p_{\mathrm{data}}\left(x\right)$ is the distribution of input data, and *z* is noise from a distribution $p_{z}\left(z\right)$. The GAN is trained in an alternative way of firstly maximizing the discriminator loss and then minimizing the generator loss. Both generator and discriminator employ independent back-propagation procedures. In this way, GANs have the ability to learn the data distribution in an unsupervised manner.

#### 3.5.2. Deep Convolutional GAN (DCGAN)

**Figure 16:**
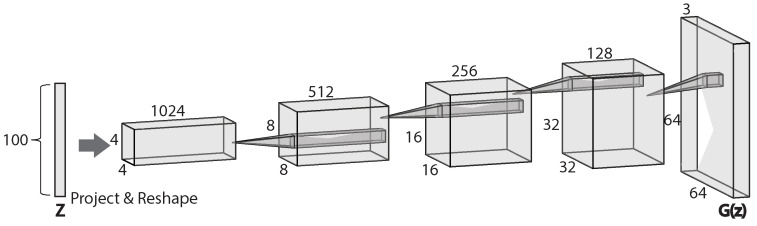
Diagram of a DCGAN.

The DCGAN, proposed by Radford et al. [114], is a convolution-based GAN. It is one of the most powerful and successful types of GAN, and has been widely used in many convolution-based generation-based techniques. Compared to GAN, the DCGAN uses convolutional and convolutional-transpose layers to implement its generator and discriminator, and this is the origin of its name. Another interesting characteristic of DCGAN is that, unlike the typical neural networks to map input to a binary output, or a regression output, or even a categorical output, the generator of a DCGAN can map from random noise to images. For example, the generator of the DCGAN in [114] takes in a noise vector of size 100×1 and maps it into an output image of size 64×64×3 (Figure 16). The DCGAN can be used to generate images as ‘real’ as possible from a distribution.

#### 3.5.3. Conditional GAN (cGAN)

**Figure 17:**
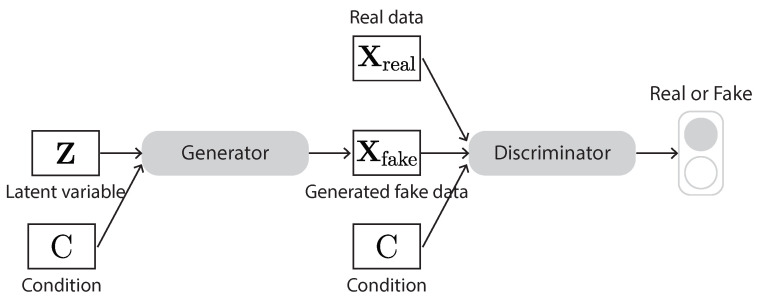
Diagram of a cGAN.

The cGAN (Figure 17) is a type of GAN whose generator and discriminator are conditioned on some auxiliary information from other modalities [115]. As a result, it can learn multimodal mapping from inputs to outputs by feeding it with different contextual information. In other words, a cGAN allows us to guide the generator to generate the kind of fake samples we want. The input to the auxiliary layer can be class labels or some other properties we expect from the generated data. As the cGAN uses some kind of labels for it to work, it is not a strictly unsupervised learning algorithm. The advantages of using additional information are (1) the convergence will be faster and (2) the generator can generate specific output given a certain label.

#### 3.5.4. Other Types of GANs

Other well-known types of GANs include Info GAN (also called iGAN) [116], Auxilary Classifier GAN (ACGAN) [117], Stacked GAN [118], Wasserstein GAN [119], Cycle GAN [120], and Progressive GAN [121].

(1) The Info GAN is a modified GAN that aims to learn interpretable and meaningful representations. To this end, it splits the input of the generator into two parts: The typical noise and a new “latent code” which is composed of control variables. The code is then made meaningful by maximizing the mutual information between the code and the generated output. This way, the generator can be trained by using the control variables to affect specific properties of the generated outputs.

(2) The ACGAN is similar to the cGAN because both their generators take noise and labels as input. However, the ACGAN has an auxiliary class label output compared to the cGAN. Therefore, the ACGAN can be seen as an extension of the cGAN. It has the effect of stabilizing the training process and allowing the generation of large, high-quality images, while learning representations in a latent space independent of class labels.

(3) The Stacked GAN is an extension of the GAN for generating images from text by a hierarchical stack of cGANs. Its architecture is composed of a set of text-conditional and image-conditional GANs. More specifically, the first-level generator is conditioned on text and generates a low-resolution image. The second-level generator is conditioned on both the text and the low-resolution image and outputs a high-resolution image.

(4) The Wasserstein GAN is an advanced GAN that aims to better approximate the distribution of data observed in a given training dataset. To this end, it uses a critic rather than a discriminator to scores the realness or fakeness of a given image. Its underlying idea is to let the generator minimize the distance between the distribution of the data in the training dataset and the distribution of the generated samples. The advantage of Wasserstein GAN is that its training process is more stable and less sensitive to model architecture and hyperparameter configurations.

(5) The Cycle GAN is an advanced GAN proposed for image-to-image translation. Its outstanding characteristic is that it learns mapping between inputs and outputs using an unpaired dataset. The Cycle GAN simultaneously trains two generators and two discriminators. One generator is responsible for generating images for the resource domain learned from, and the other is responsible for generating images for the target domain. Each generator has a corresponding discriminator.

(6) The Progressive GAN is proposed for stable training and large-scale high-resolution image generation. Similar to a GAN, the Progressive GAN consists of a generator and a discriminator, which are symmetrical to each other. Its key feature is to progressively grow the generator and discriminator, starting from a low resolution, and then adding new layers to increase the model’s fine details as training progresses. As a result, training is faster and more stable, producing images of unprecedented quality.

### 3.6. Graph Neural Network (GNN)

Graph neural networks are a class of neural networks that operate on the graph structure, where data are generated from non-Euclidean domains and represented as graphs with complex relationships and interdependencies between nodes [122]. Examples of graph data include social networks, citation networks, molecular structures, and many other types of data that are organized in a graph format.

A graph is represented as G=(V,E), where *V* is the set of vertices or nodes, and *E* is the set of edges. Let $v_{i}\in V$ denote a node and $e_{ij}=\left(v_{i},v_{j}\right)\in E$ denote an edge pointing from $v_{i}$ to $v_{j}$. The neighborhood of a node *v* is defined as N(v)={u∈V|(v,u)∈E}. The adjacency matrix A is an n×n matrix with $A_{ij}=1$ if $e_{ij}\in E$ and $A_{ij}=0$ if $e_{ij}\notin E$. A graph may have node attributes X, where $X\in R^{n\times d}$ is a node feature matrix with $x_{v}\in R^{d}$ representing the feature vector of a node *v*. Furthermore, a graph may have edge attributes $X^{e}$, where $X^{e}\in R^{m\times c}$ is an edge feature matrix with $x_{v,u}^{e}\in R^{c}$ representing the feature vector of an edge (v,u). A directed graph is a graph with all edges directed from one node to another. An undirected graph is considered as a special case of directed graphs, where there is a pair of edges with inverse directions if two nodes are connected. A graph is undirected if and only if the adjacency matrix is symmetric. A spatial–temporal graph is an attributed graph where the node attributes change dynamically over time. The spatial–temporal graph is defined as $G^{\left(t\right)}=\left(V,E,X^{\left(t\right)}\right)$ with $X^{\left(t\right)}\in R^{n\times d}$.

There are three general types of analytics tasks on graphs: graph-level, node-level, and edge-level. In a graph-level task, the goal is to predict a single property for an entire graph [123]. This is often referred to as a graph classification task, as the entire graph is associated with a label. To obtain a compact representation on the graph level, GNNs are often combined with pooling and readout operations [124,125,126]. Node-level tasks are concerned with predicting the identity or role of each node in a graph [127], and therefore, the model outputs relate to node regression and node classification tasks. Recurrent GNNs and convolutional GNNs can extract high-level node representations by information propagation and graph convolution. With a multiperceptron or a softmax layer as the output layer, GNNs are able to perform node-level tasks in an end-to-end manner. Similarly, an edge-level task predicts the property or presence of edges in a graph, hence the outputs relate to the edge classification and link prediction tasks. With two nodes’ hidden representations from GNNs as inputs, a similarity function or a neural network can be utilized to predict the label/connection strength of an edge.

Based on the model architectures, GNNs can be categorized into recurrent graph neural networks, convolutional graph neural networks, graph autoencoders and generative graph neural networks, and spatial-temporal graph neural networks.

#### 3.6.1. Recurrent Graph Neural Network (RecGNN)

RecGNNs aim to learn node representations with recurrent architectures. A representative model in this class is the GNN proposed by Scarselli et al. [128], which updates the states of nodes by exchanging neighborhood information recurrently until a stable equilibrium is researched, as in the following equation:

(10) $$h_{v}^{\left(t\right)}=\sum_{u\in N(v)}f\left(x_{v},x_{\left(v,u\right)}^{e},x_{u},h_{u}^{\left(t-1\right)}\right),$$

where f(·) is the parametric function and $h_{v}^{\left(0\right)}$ is the initial state randomly set. Other popular RecGNNs include the GraphESN [129] which extends echo state networks to improve the training efficiency of GNN, and the Gated GNN [130] which employs a gated recurrent unit as the recurrent function that reduces the recurrence to a fixed number of steps. RecGNNs are conceptually important and inspired later research on ConvGNNs. In particular, the idea of information passing is inherited by spatial-based ConvGNNs.

#### 3.6.2. Convolutional Graph Neural Network (ConvGNN)

ConvGNNs generalize the operation of convolution from grid data to graph data. The main idea is to generate a representation of a node *v* by aggregating its own features $x_{v}$ and neighbors’ features $x_{u}$, where u∈N(v). Different from RecGNNs, ConvGNNs stack multiple graph convolutional layers to extract high-level node representations. ConvGNNs play a central role in building up a great deal of other complex GNN models. ConvGNNs can be further divided into spectral-based methods and spatial-based methods: The first category defines graph convolutions by introducing filters from the perspective of graph signal processing [131], and the latter inherits ideas from RecGNNs to define graph convolutions by information propagation.

Spectral-based methods have a solid mathematical foundation in graph signal processing, and they are based on the normalized graph Laplacian matrix which is a mathematical representation of an undirected graph, defined as $L=I_{n}-D^{-1/2}AD^{-1/2}$, where D is a diagonal matrix of node degrees. This normalized Laplacian matrix can be factored as $L=U\Lambda U^{T}$, where Λ and U denote the ordered diagonal matrix of eigenvalues and the corresponding eigenvector matrix, respectively. The graph convolution of an input signal x with a filter $g\in R^{n}$ is then defined as:

(11) $$x{*}_{G}g=F^{-1}\left(F\left(x\right)⊙F\left(g\right)\right)=U\left(U^{T}x⊙U^{T}g\right)$$

where ⊙ denotes the element-wise product, and F(x) is the graph Fourier transform of the signal x. Let $g_{\theta }=\mathrm{diag}\left(U^{T}g\right)$ denote a filter, the spectral graph convolution is simplified as:

(12) $$x{*}_{G}g_{\theta }=Ug_{\theta }U^{T}x.$$

Popular spectral-based GNNs inlcude the Spectral CNN [132], ChebNet [125] and GCN [127], where the key difference lies in the design of the filter $g_{\theta }$.

The spatial-based graph convolution is defined on the nodes’ spatial relations, and it convolves a node’s representation with its neighbors’ representations to derive the updated representation, inheriting the idea of information propagation of RecGNNs. Representative spatial-based GNNs include the Diffusion CNN [133], message-passing neural network (MPNN) [134], GraphSage [135], and graph attention network (GAT) [136] (which brings in attention mechanisms), mixture model network (MoNet) [137], and FastGCN [138]. Since GCN [127] bridged the gap between spectral-based approaches and spatial-based approaches, spatial-based methods have developed rapidly recently due to their attractive efficiency, flexibility, and generality.

#### 3.6.3. Graph Autoencoder (GAE) and Other Generative Graph Neural Networks

GAEs and generative GNNs are unsupervised learning frameworks that encode nodes into a latent vector space and decode graph information from the latent representations. GAEs are used to learn network embeddings and graph generative distributions. A network embedding is a low-dimensional vector representation of a node that preserves a node’s topological information. For network embedding, GAEs learn latent node representations through reconstructing graph structural information, such as the graph adjacency matrix. Representative GAEs for network embedding include the DNGR [123], SDNE [139], GAE [140], Variational GAE [140], and GraphSage [135]. These models combine different AEs and other models such as ConvGNNs and LSTM. With multiple graphs, GAEs are able to learn the generative distribution of graphs by encoding graphs into hidden representations and decoding a graph structure given hidden representations. The majority of GAEs for graph generation are designed to solve the molecular graph generation problem [141], which has a high practical value in drug discovery. These methods either propose a new graph sequentially, such as DeepGMG [142] and GraphRNN [143], or in a global manner, such as GraphVAE [144]. GNNs are also integrated with the architecture and training strategy of GANs, resulting in MolGAN [145] and NetGAN [146].

#### 3.6.4. Spatial–Temporal Graph Neural Network (STGNN)

Graphs in many real-world applications are dynamic, both in terms of graph structures and graph inputs. STGNNs occupy important positions in capturing the dynamics of graphs. The task of STGNNs can be forecasting future node values or labels, or predicting spatial–temporal graph labels. STGNNs capture spatial and temporal dependencies of a graph simultaneously. Current approaches integrate graph convolutions to capture spatial dependence with RNNs or CNNs to model temporal dependence. Most RNN-based approaches capture spatial–temporal dependencies by filtering inputs and hidden states passed to a recurrent unit using graph convolutions [147]. As alternative solutions, CNN-based approaches tackle spatial–temporal graphs in a non-recursive manner with the advantages of parallel computing, stable gradients, and low memory requirements. CNN-based approaches interleave 1-D-CNN layers with graph convolutional layers to learn temporal and spatial dependencies, respectively, as in the CGCN [148].

#### 3.6.5. Training of GNNs

Given a single network with part of the nodes labeled and others unlabeled, ConvGNNs can be trained in a semi-supervised manner to learn a robust model that effectively identifies the class labels for the unlabeled nodes [127]. To this end, an end-to-end framework can be built by stacking a couple of graph convolutional layers followed by a softmax layer for multiclass classification. In addition, GNNs can be trained in a supervised manner for graph-level classification, which is achieved by applying the graph pooling layers and readout layers [123]. Finally, GNNs can learn graph embedding in a purely unsupervised manner in an end-to-end framework (e.g., an AE framework [140]).

### 3.7. Transformer

The transformer [149] is a prominent type of deep learning models that has achieved impressive advances on various tasks such as computer vision and audio processing. Originally proposed for natural language processing, the transformer mainly relies on deep neural networks and the self-attention mechanism, emphasizing the global dependencies between the input and output, thereby providing strong representation capability and state-of-the-art performance. Due to the significant improvement made by the transformer model, several variants have been proposed for either improving model performance or adapting the model to specific tasks in recent years.

#### 3.7.1. Vanilla Transformer

**Figure 18:**
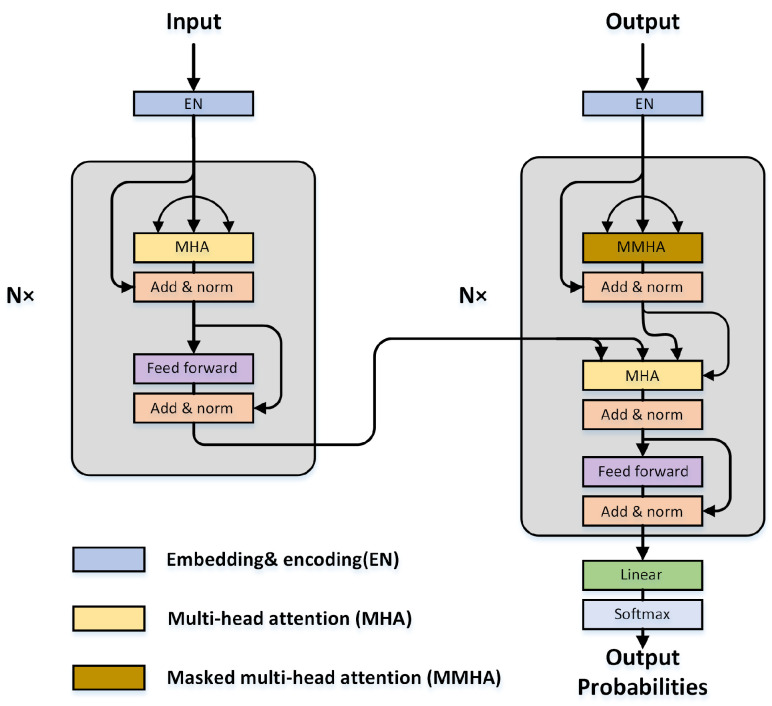
The model architecture of the Transformer.

The transformer follows the encoder-decoder structure (Figure 18). The encoder is composed of a stack of identical blocks with two modules: The multihead self-attention layers and the position-wise fully connected feed-forward network (FFN). A residual skip connection, followed by a batch normalization layer, is applied to each submodule. Besides the two modules in the encoder block, the decoder block inserts an additional masked multihead attention layer, which is specially modified to avoid positions from attending to subsequent positions. In the following, we introduce the two modules in more detail.

(1) The multihead attention layer adopts the self-attention mechanism with the Query-Key-Value (Q-K-V) model. The inputs are first projected into three kinds of vectors: The query vector q, the key vector k with dimension $d_{k}$, and the value vector v with dimension $d_{v}$. After packing a set of these vectors together into three matrices, namely queries $Q\in R^{N\times D_{k}}$, keys $K\in R^{N\times D_{k}}$, and values $V\in R^{N\times D_{v}}$, the scale dot-product attention can be computed as follows:

(13) $$\mathrm{Attention}\left(Q,K,V\right)=\mathrm{softmax}\left(\frac{Q\cdot K^{⊤}}{\sqrt{d_{k}}}\right)\cdot V=AV.$$

In this process, $Q\cdot K^{⊤}$ computes a score between each pair of input vectors and yields the degree of attention. The produced scores are divided by $\sqrt{d_{k}}$ to avoid the vanishing gradient problem and improve the stability of training. The softmax operator transforms the divided scores into probabilities A, which is also called the attention matrix. After multiplying values V with the attention matrix, vectors with higher probabilities receive more attention from the subsequent layers.

Rather than using a single self-attention operation, multihead attention learns *h* different linear projections and transforms the queries, keys, and values into *h* sets with $D_{k},D_{k},D_{v}$ dimensions. Then, the self-attention operation can be implemented in parallel and produce different output values, which are subsequently concatenated and projected linearly back to $D_{m}$-dimension feature.

(14) $$\begin{array}{cc} \mathrm{Multihead}(Q,K,V) & =\mathrm{concat}\left({\mathrm{head}}_{1},\ldots ,{\mathrm{head}}_{h}\right)W^{O}\\ \mathrm{where}\;{\mathrm{head}}_{i} & =\mathrm{Attention}\left(QW_{i}^{Q},KW_{i}^{K},VW_{i}^{V}\right) \end{array}$$

where $W_{i}^{Q}\in R^{D_{\mathrm{model}\;}\times D_{k}}$, $W_{i}^{K}\in R^{D_{\mathrm{model}\;}\times D_{k}}$, $W_{i}^{V}\in R^{D_{\mathrm{model}\;}\times D_{v}}$ denote the parameters for linear projections for the *Q*, *K*, *V* branches, respectively. $W_{i}^{O}\in R^{hD_{v}\times D_{\mathrm{model}\;}}$ denote the parameters for linear projections after concatenation. In the vanilla transformer, $D_{k}=D_{v}=D_{\mathrm{model}\;}/h=64$ and h=8.

(2) The fully connected feed-forward network consists of two linear transformations with a RelU activation function in between.

(15) $$\mathrm{FFN}\left(x\right)=\max\left(0,xW_{1}+b_{1}\right)W_{2}+b_{2}$$

#### 3.7.2. Transformer Variants

Motivated by the impressive success of the transformer, researchers have devoted numerous efforts to make further progress in a variety of tasks. Improvements have been achieved from three perspectives: using pretrained models (PTM), modifying the vanilla transformer architecture, and adapting to new tasks.

(1) Using pretrained models: Compared with training a model from scratch, using pretrained transformer models has been revealed to be beneficial for building up universal feature representations. Powerful PTMs help reduce the need for task-specific architectures by simple fine-tuning on the downstream datasets. Bidirectional Encoder Representations from Transformers (BERT) [150] is the first fine-tuning based model with transformer architecture for natural language understanding and pushed the performance frontier of 11 NLP tasks. Generative Pretrained Transformer (GPT) series [151,152] show that massive PTMs with large-scale parameters can help achieve strong universal representation ability and provide state-of-the-art performance on different types of tasks, even without the fine-tuning process. Bidirectional and Auto-Regressive Transformers (BART) [153] generalized the pretraining scheme and built a denoising auto-encoder model to further boost the capacity in language understanding.

(2) Modifying the vanilla transformer architecture: As self-attention is considered to be the fundamental component of the transformer, various architecture modifications have been proposed to address its limitations including computational complexity and ignorance of prior knowledge. Representative modifications including Low-rank based Sparse attention [154], linearized attention [155], improved multihead attention [156], and prior attention [157] have been designed to reduce complexity and make the most of the structural prior. Another branch of important modifications is adapting the architecture to be lightweight in terms of model size and computation, such as Lite Transformer [158], Funnel Transformer [159] and DelighT [160].

(3) Adopting to new tasks: Besides NLP, the transformer concept has been adapted in various fields, including computer vision [161,162,163,164,165,166] and multimodal data processing. For vision tasks, the transformer architecture has been extensively explored. ViT [161] is the first vanilla transformer architecture applied to image classification tasks without any alternation. It directly reshapes the image patches and flattens them into a sequence as the input. Experiments on large datasets such as ImageNet and JFT-300M show that the transformer has great potential in capturing long-range dependency and suits vision tasks well. Researchers also attempted to modify the network architecture and make it more feasible to vision tasks. Transformer in Transformer (TNT)[165], iGPT [162], and Swin Transformer [166] are representative models in this regard.

### 3.8. Bayesian Neural Network (BNN)

While DNNs have been shown to achieve great success in different applications, they are unable to deal with the uncertainty of a given task due to model uncertainty. This is due to their essence of using BP to approximate a minimal cost of point estimates of the network parameters, while discarding all other possible parametrizations of the network [167]. The BNN is proposed to mitigate this by providing a strict framework to train an uncertainty-aware neural network [168,169]. The application domains of BNN are very wide, including recommender systems [170], computer vision [171], natural language processing [172], speech recognition [173], biomedical applications [174], and so on.

The BNN is essentially a stochastic neural network trained using a Bayesian method [175,176]. A stochastic neural network is a type of DNN involving stochastic components into its network. The stochastic component is used to simulate multiple possible models with their associated probability distribution. The main aim of a stochastic neural network is to obtain a better idea of the uncertainty associated with the model. This is achieved by comparing the predictions of multiple models obtained by sampling the model parameterization. The uncertainty is low if these models generate consistent predictions, otherwise the uncertainty is high. This process can be formulated as:

(16) $$y=\Phi_{\theta }\left(x\right)+\varepsilon ,$$

where θ=(W,b) are the parameters of the neural network which follow the probability distribution p(θ), and ε is the random noise used to ensure the function Φ represented by the network is only an approximation. This way, a BNN can be defined as a stochastic neural network trained using Bayesian inference [177].

The uncertainty of a neural network is a measure of how certain a model is with its prediction. With BNNs, there are two kinds of uncertainty: aleatoric uncertainty and epistemic uncertainty. The aleatoric uncertainty refers to the noise inherent in the observations, and cannot be reduced by collecting more data. The epistemic uncertainty is also known as model uncertainty and is caused by the model itself. It can be reduced by collecting more data. The BNN usually solves this issue by placing a probability distribution on the network weights or by learning a mapping from input to probabilistic outputs to derive the estimation of uncertainty. More specifically, the epistemic uncertainty is modeled by placing a prior distribution on the network weights and then capturing the degree of change of these weights over the data. The aleatoric uncertainty is modeled by placing a distribution on the outputs of the model.

One problem of BNNs is that they are hard to train. In practice, the Bayes by Backprop algorithm proposed by Blundell et al. [178] is used for learning a probability distribution on the network weights. Another problem of using BNNs is that they rely on prior knowledge, and it is challenging to derive insights about plausible parametrization for a given model before training. However, BNNs have become promising due to the following advantages. Firstly, thanks to its stochastic component, BNNs can quantify uncertainty, which means the uncertainty is more consistent with the observed errors. Moreover, BNNs are very data-efficient because they can learn from a small dataset without overfitting. This is due to the fact that they can distinguish the epistemic and aleatoric uncertainty. Finally, BNNs enable the analysis of learning methods, which is important for many fields such as traffic monitoring and medicine.

### 3.9. Fuzzy Deep Neural Networks (FDNN)

#### 3.9.1. Introduction of FDNN

Typical DNNs are trained by minimizing the loss or error given an input through gradient descent-based weight update [179]. This is a calculus-based method that iteratively computes the minimum of the error function. However, obvious disadvantages of this method are that it is computationally intensive and may not find the global minimum [180]. To address this issue, multiple FDNNs have been proposed, for example, the fuzzy RBM [181] and the Takagi Sugeno fuzzy deep network [182]. As an emerging method, FDNNs have been applied in distributed systems [183], cloud computing [184], traffic control [185], healthcare [186], image processing [187], and various other areas.

A FDNN is a hybridization of DNNs and fuzzy logic methods, to solve various complex problems involving high-dimensional data. The key benefit of a DNN is its ability to learn from data, but it cannot clarify how its final output is achieved. Combined with fuzzy logic, a FDNN can interpret the results generated by the network [188]. More specifically, a FDNN introduces an additional fuzzy inference into a DNN to create an explainable rule-based structure. This way, through this rule-based structure, how a decision is made by the network is understandable.

#### 3.9.2. Types of FDNN

A FDNN can be comprised by a broad category of DNNs and fuzzy inference systems in different architectures. Current architectures in the literature can be classified into three categories: sequential FDNN, parallel FDNN, and cooperative FDNN [189].

**Figure 19:**
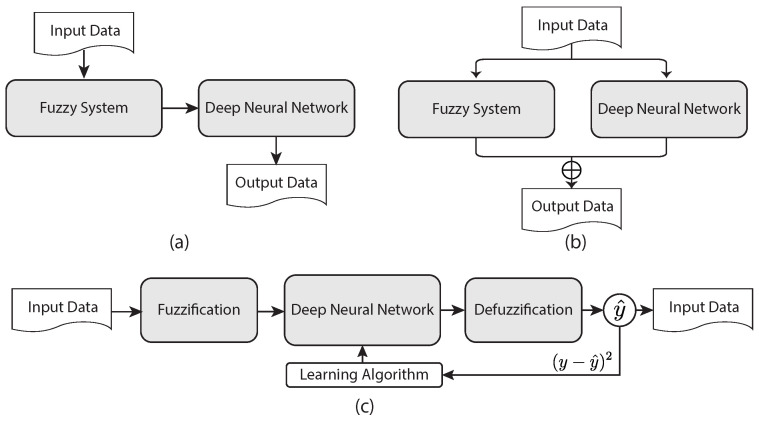
Diagram of three types of FDNN. (**a**) Sequential FDNN, (**b**) Parallel FDNN, and (**c**) Cooperative FDNN.

A sequential FDNN has a structure that passes the data through the DNN and the fuzzy inference system sequentially (Figure 19a). It is suitable for solving problems involving high linearity, such as text documents, time-series data, video classification, and speech recognition.

A parallel FDNN has a structure that passes the data separately through the DNN and the fuzzy inference system, and fuses the results to generate the output (Figure 19b). This kind of FDNN has been used for multiple classification tasks [190].

A cooperative FDNN has a structure where the input data are firstly passed through a fuzzy interface block to generate fuzzy values, which are subsequently input to a DNN followed by a defuzzification block to convert the fuzzy values into output data (Figure 19c). An example application of the cooperative FDNN is fuzzy classification [191].

### 3.10. Deep Reinforcement Learning (DRL)

A reinforcement learning (RL) agent executes a sequence of actions and observes states and rewards, with major components being the value function, policy and model. A RL problem may be formulated as a prediction, control or planning problem, and solution methods may be model-free or model-based, with value function and/or policy [192]. Exploration-exploitation is a fundamental trade-off in RL. Knowledge would be critical for RL. DRL, integrating deep learning and RL, represents a step forward in building autonomous systems with a higher-level understanding.

#### 3.10.1. Deep Q-Network

**Figure 20:**
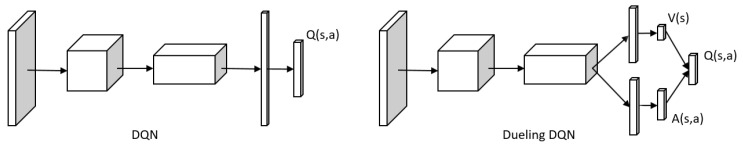
Deep Q-Network and Dueling Deep Q-Network architectures.

Value function is a fundamental concept in reinforcement learning, and temporal difference learning [193] and its extension, Q-learning [194], are classic algorithms for learning state and action value functions respectively. Q-learning learns the action-value function Q(s,a), i.e., how good it is to take an action *a* at a particular state *s*, to build a memory table Q[s,a] that stores *Q* values for all possible combinations of *s* and *a*. However, if the combinations of states and actions are large, the memory and computation requirement for *Q* is very high. Deep Q-learning addresses this problem by generalizing the approximation of the *Q*-value function rather than remembering the solutions. The challenge in RL is that both the input and target change constantly during the process, which makes training unstable. Deep Q-Network (DQN) [195] ignited the field of DRL, making an important contribution in stabilizing the training of action value function approximation with DNNs using experience replay. In addition, it designs an end-to-end RL approach, with only the pixels and the game score as inputs, so that only minimal domain knowledge is required. Important extensions of DQN are the Double DQN [196] which addresses the over-estimate issue in Q-learning, and Dueling DQN [197] which uses two separate heads to compute the state value function V(s) and associated advantage function A(s,a) (Figure 20).

#### 3.10.2. Asynchronous Advantage Actor-Critic (A3C)

A3C [198] uses multiple agents with each agent having its own network parameters and a copy of the environment. These agents interact with their respective environments asynchronously, learning with each interaction. Each agent is controlled by a global network. As each agent gains more knowledge, it contributes to the total knowledge of the global network. The presence of a global network allows each agent to have more diversified training data. An actor-critic algorithm predicts both the value function V(s) and the optimal policy function π(s). The learning agent uses the value of the value function (critic) to update the optimal policy function (actor). It determines the conditional probability P(a|s;θ), the parameterized probability that the agent chooses the action *a* when in state *s*. Different from most deep learning algorithms, asynchronous methods can run on a single multi-core CPU.

#### 3.10.3. Trust Region Policy Optimization (TRPO)

A policy maps the state to action. Policy optimization is to find an optimal mapping from state to action. Policy gradient methods are popular in RL. The basic principle uses gradient ascent to follow policies with the steepest increase in rewards. However, large policy changes can destroy training, and it is not easy to map changes between policy and parameter space and to deal with the vanishing or exploding gradient problems and poor sample efficiency. The challenge is to have an accurate optimization method to limit the policy changes and guarantee any change will lead to improvement in rewards. A more commonly used method is to use a trust region, in which optimization steps are restricted to lie within a region where the approximation of the true cost function still holds. By preventing updated policies from deviating too wildly from previous policies, the chance of a catastrophically bad update is lessened, and many algorithms that use trust regions guarantee or practically result in monotonic improvement in policy performance. The idea of constraining each policy gradient update, as measured by the Kullback–Leibler (KL) divergence between the current and proposed policy, has a long history in RL [199]. TRPO [200] is an algorithm in this line of work that has been shown to be relatively robust and applicable to domains with high-dimensional inputs. To achieve this, TRPO optimizes a surrogate objective function—specifically, it optimizes an (importance sampled) advantage estimate, constrained using a quadratic approximation of the KL divergence. It avoids parameter updates that change the policy too much with a KL divergence constraint on the size of the policy update at each iteration. The generalized advantage estimation (GAE) proposed several more advanced variance reduction baselines [201]. The combination of TRPO and GAE remains one of the state-of-the-art RL techniques in continuous control.

### 3.11. Deep Transfer Learning (DTL)

Deep learning has a strong dependence on massive training data compared to traditional machine learning methods. Having sufficient training data is a prerequisite for a deep learning model to understand the latent patterns of the data. However, this is quite a challenge itself since the collection of data is time consuming and expensive. It is difficult to build a large-scale and high-quality annotated dataset in many fields. In addition, the training of deep learning models relies on intensive computation, which in practice can be challenging due to limited resources (e.g., high performance GPUs) and time constraints. Transfer learning is a concept of reusing a pretrained model on a new problem, which is an efficient way to tackle the insufficient training data problem and reduce the computational resource requirement and training time. It is very common in deep learning to use a pretrained model as a feature extractor in a new task or fine-tune the pretrained model (or some high-level parts of the model) to a new learning task.

Let $D_{s}$ and $D_{t}$ denote the source domain and target domain, and $T_{s}$ and $T_{t}$ denote two learning tasks ($D_{s}\neq D_{t}$ or $T_{s}\neq T_{t}$), respectively. Transfer learning can be defined as the process of enhancing the learning of the target predictive function $f_{T}\left(\cdot \right)$ in $D_{t}$ using knowledge derived from $D_{s}$ and $T_{s}$. It is a deep transfer learning task when $f_{T}\left(\cdot \right)$ is a nonlinear function that reflects a DNN. There are three forms of transfer learning: inductive transfer learning [202], transductive transfer learning [203], and unsupervised transfer learning [204]. In the first, $T_{s}$ and $T_{t}$ are different, and some labelled data in $D_{t}$ are required to induce $f_{T}\left(\cdot \right)$ for use in $D_{t}$. In the second, we have the same $T_{s}$ and $T_{t}$ but different $D_{s}$ and $D_{t}$, while no labelled data in $D_{t}$ are available but labelled data in $D_{s}$ are available. Finally, in the last setting, $T_{t}$ is different from but related to $T_{s}$, and there are no labelled data in both $D_{s}$ and $D_{t}$ during training. The focus is on solving unsupervised learning tasks in $D_{t}$, such as clustering, dimensionality reduction, and density estimation.

According to the content to be transferred, transfer learning methods can be categorised into four cases: (1) instance-based approaches try to reweight the samples in $D_{s}$ for learning in $D_{t}$ [205]; (2) feature-based approaches encode knowledge into feature representations which are transferred across domains to help improve the performance of $T_{t}$ [202]; (3) parameter-based approaches transfer knowledge across tasks through the shared parameters of the $D_{s}$ and $D_{t}$ learning models [206]; and (4) relational-based approaches, which transfer the knowledge through learning the common relationships between $D_{s}$ and $D_{t}$. Recently, statistical relational learning techniques dominate this context [207].

### 3.12. Federated Learning (FL)

FL is applied in a situation where a group of clients wants to collaboratively train a global model without sharing their private local dataset [208]. Compared with conventional machine learning methods which require gathering different datasets, clients in FL collaboratively train a global model by exchanging local model weights/gradients without sharing their local dataset. There are typically two key players in FL: (1) the clients holding the local dataset and training the local model, and (2) the central server coordinating the training process and updating the global model. In general, FL contains three phases [209]:

Phase 1: FL initialization. The central server initializes the FL training model and sets the hyperparameters, including the number of FL training iterations, the total number of participating clients, the number of clients selected at each training iteration, and the local batch size used in each training iteration. Then, the central server broadcasts the global model to the selected clients.
Phase 2: Local model training and updating. In each FL training iteration, clients first update the local model using the shared global model and train the local model using the local dataset. Then, clients send the local model weights or gradients to the central server for model aggregation.
Phase 3: Global model aggregation. The central server aggregates the model weights or gradients from the participating clients and shares the aggregated model to the clients for the next training iteration.

Algorithm 1 shows the pseudocode of an FL system proposed in [210]. According to the characteristics of training data, FL methods are usually classified into two categories: horizontal FL and vertical FL [208,211].
**Algorithm 1** FedAvg [210]**Input:**  $N_{\mathrm{global}}$: Maximum number of global iterations, *n*: The total number of participating clients, *m*: The number of clients used in each global iteration, $N_{\mathrm{local}}$: The number of local epochs, and η: The local learning rate.**Output:**  Global model weight $w_{G}$**Processing:**1: [*Central Server*]2: Initialize $w_{G}^{0}$3: **for** each iteration *t* from 1 to $N_{\mathrm{global}}$ **do**4:  $M_{t}$ includes *m* clients randomly selected from the *n* clients5:  **for** each client $i\in M_{t}$ **in parallel do**6:   $w_{i}^{t},N_{i}\leftarrow \mathrm{LocalTraining}\left(i,w_{G}^{t}\right)$7:  **end for**8:  $w_{G}^{t+1}=\frac{1}{\sum_{j=1}^{m}N_{j}}\sum_{i=1}^{m}N_{i}w_{i}^{t}$9: **end for**10: [*Each Participating Client*]11: LocalTraining(i,w):12: $B_{i}$ is the set of batches for the local dataset $D_{i}$13: **for** each epoch *j* from 1 to $N_{\mathrm{local}}$ **do**14:  **for** each batch $b\in B_{i}$ **do**15:   w←w−η∇L(w;b)16:  **end for**17: **end for**18: **return** the weights w and $N_{i}=\left|D_{i}\right|$

#### 3.12.1. Horizontal FL (HFL)

HFL is used in scenarios where the datasets of the clients share the identical feature space but a different sample ID space [210,212]. For example, the electricity usage held by different electricity supplier companies may have the same feature space but different ID space. The communication protocols in FL can be divided into two classes: client-server protocol [210,213] and peer-to-peer protocol [212,214,215]. The client-server protocol deploys a central server to coordinate the training process, whereas the peer-to-peer protocol randomly selects a client as the server for the coordination work in each iteration.

In the client-server protocol, the clients are assumed honest and the server is assumed honest but curious. To avoid private information leakage, the exchanged model parameters are usually encrypted or masked by clients. The key steps are summarized as follows:

Step 1: The central server initializes the model and hyperparameters and allocates computation tasks to named clients.
Step 2: The participating clients train their local models on their local dataset, encrypt the model weights/gradients, and transmit them to the central server.
Step 3: The server conducts model aggregation, for example by averaging.
Step 4: The server broadcasts the updated model to all clients.
Step 5: The clients decrypt the model and update their local models.

In the peer-to-peer protocol, as there is no central server, two approaches are usually adopted to coordinate the training process:

(1) Cyclic Setting: All clients form a circular chain, denoted by $\left\{C_{1},C_{2},\ldots ,C_{n}\right\}$. Client $C_{i}$ transmits its local model to client $C_{i+1}$. Client $C_{i+1}$ aggregates the received model with its local model which is trained on its local dataset and then transmits the updated model along the chain to client $C_{i+2}$. The training process stops once the termination condition is met.
(2) Random Setting: Client $C_{t}$ randomly picks a client $C_{i}$ from all participants with equal chance and sends its model information to another client $C_{i}$. $C_{i}$ aggregates the received model with its local model which is trained on its local dataset, then randomly picks another client $C_{j}$ with equal chance and sends the updated model to it. The training process stops once the termination condition is met.

#### 3.12.2. Vertical FL (VFL)

VFL is used in scenarios where datasets between participating clients share the identical sample ID space but a different feature space. For example, a bank and an online shopping company may have the same customers but provide different services. The communication protocols for VFL can be divided into two classes: communication with a third-party coordinator [216] and communication without a third-party coordinator [217]. Assume that two clients, $C_{1}$ and $C_{2}$, plan to train a global model using their local datasets, and that samples from $C_{1}$ are labeled. In addition, $C_{1}$ and $C_{2}$ are assumed honest but curious to each other.

To protect the private data, the communication protocol with a third-party coordinator is designed as follows [216]:

Step 1: As the two datasets of $C_{1}$ and $C_{2}$ contain samples with different IDs, it is necessary to extract the common samples sharing the same IDs [218].
Step 2: The coordinator $C_{3}$ produces a pair of public and private keys and broadcasts the public key to $C_{1}$ and $C_{2}$.
Step 3: $C_{1}$ and $C_{2}$ compute encrypted gradients and add a mask. In addition, $C_{1}$ computes the encrypted loss. $C_{1}$ and $C_{2}$ then transmit the encrypted results to $C_{3}$.
Step 4: $C_{3}$ decrypts the received results and broadcasts them back to $C_{1}$ and $C_{2}$. $C_{1}$ and $C_{2}$ then update their local model using the received information.

To protect the private data, the communication protocol without a third-party coordinator is designed as follows [217]:

Step 1: A sample ID alignment process [219] is first employed to select the shared IDs between $C_{1}$ and $C_{2}$. Samples sharing the same IDs are confirmed to train a vertical FL model.
Step 2: $C_{1}$ produces an encryption key pair and transmits its public key to $C_{2}$.
Step 3: The two clients initialize their model weights and compute their partial prediction results. $C_{2}$ then transmits its result to $C_{1}$.
Step 4: $C_{1}$ computes the model residual, encrypts the residual, and transmits it to $C_{2}$.
Step 5: $C_{2}$ computes the encrypted gradient and transmits the masked gradient to $C_{1}$.
Step 6: $C_{1}$ decrypts the masked gradient and transmits it back to $C_{2}$. Then, $C_{1}$ and $C_{2}$ update their model locally.

### 3.13. Multiple Instance Learning (MIL)

#### 3.13.1. Introduction of MIL

The concept of multiple instance learning was firstly proposed by Dietterich et al. [220] for investigating the problem of drug activity prediction. It is a type of weakly supervised learning where the training set is composed of many bags and each bag contains many instances, and a label is provided for the entire bag rather than each individual instance in it. This problem occurs when dealing with a lack of detailed annotation for large quantities of data. For example, it emerges when developing computer-aided diagnosis algorithms where medical images have only a patient-level diagnosis label rather than costly local labels annotated by experts [221]. Furthermore, it naturally occurs in a number of real-world learning scenarios, including image and video classification [222], document classification [223], and sound classification [224].

Generally, there are two assumptions in multiple instance learning: The standard and the collective assumption. The former assumes only negative instances are contained in negative bags, while one or more positive instances are contained in positive bags. This means that as long as there is one positive instance in the bag, the bag is positive. On the contrary, the collective assumption refers to cases where more than one positive instance is needed to identify a positive bag. These two assumptions are applied to different problem domains. For example, the standard assumption works well for drug activity prediction, while the collective assumption is more suitable for traffic jam detection.

#### 3.13.2. Training Mechanism of MIL

The MIL problem under the standard assumption can be solved through alternate optimization. Specifically, the labels of all instances are assumed to be known at first, then a classification model can be obtained through supervised learning. Subsequently, this model is used to make predictions for each training instance, and the labels of the training instances are updated accordingly, and then this classification model can be retrained with the updated labels again, and this process repeats until convergence. Thus, the optimization process has mainly two parts: supervised learning and label updating.

When training the supervised learning model, only the predicted “most correct” (i.e., the highest classification score) is selected from the positive instance bag, and other instances in the positive instance bag are discarded, regardless of whether the prediction is positive. This is because, under the standard assumption, the MIL can only consider the “most correct” instance in the positive instance bag. Therefore, this selection strategy is exactly in line with the problem definition. In addition, if there are enough negative instances, only the instance that is predicted to be “most correct” in each negative instance bag can be used for training. Such a negative instance is also called hard instance or most violated sample. In practice, they are most effective for fast model convergence.

#### 3.13.3. Challenges of Using MIL

The unique challenges of using MIL arise from four aspects: The level of prediction, the composition of bags, the ambiguity of instance labels, and the distribution of the data [225]. These factors affect the choice and the performance of MIL algorithms.

(1) The level of prediction refers to whether a network makes the prediction on a bag-level or an instance-level. These two kinds of tasks employ different loss functions, and thus algorithms designed for bag classification are not optimal for instance classification. Cheplygina et al. [226] details how to choose algorithms for different problems.
(2) The composition of bags refers to the ratio of instances from each class or the relation between instances. The proportion of positive instances in positive bags is generally defined as witness rate (WR). If the WR is very high, which means positive bags contain only a few negative instances, the problem can be solved in a regular supervised framework. However, if the WR is very low, which means a serious class imbalance problem because a few positive instances have a limited effect on training the network, many algorithms will have a poor performance. Several MIL algorithms have been proposed for this problem [227,228,229].
(3) The ambiguity of instance labels refers to label noise or instances not belonging to a class clearly. This is inherent to weakly supervised learning. Some MIL algorithms impose strict requirements on the correctness of bag labels, such as the DD algorithm [230]. For practical problems where positive instances may be found in negative bags, algorithms working under the collective assumption are needed [231].
(4) The distributions of positive and negative instances also affect MIL algorithms. This has two sides. First, the positive instances can either be located in a single cluster in feature space or be corresponding to many clusters, which leads to different applicable MIL algorithms [230,232]. Second, the distribution of the training data can or cannot entirely represent the distribution of negative instances in the test data, which also leads to different applicable MIL algorithms [233,234].

## 4. Deep Learning in Diverse Intelligent Sensor Based Systems

### 4.1. General Computer Vision Sensor Systems

A well-known application domain of deep learning is general computer vision, where the processed data are images and videos acquired from camera-based sensor systems. The research in this domain focuses on enabling computers to gain an understanding like that of human vision from images or videos. Deep learning is used for a wide range of important tasks in this domain, as described next.

#### 4.1.1. Image Classification

Conceptually, image classification is one of the simplest yet most fundamental problems in computer vision. It refers to the process of predicting information classes from an image. CNNs are the most commonly employed techniques for solving this problem. Specifically, the CNNs take an image as input and aim to output the class of the input image. Since AlexNet [37] achieved remarkable classification performance in the ImageNet challenge, many types of CNN models have been proposed for image classification, such as VGG [39], ResNet [41], and DenseNet [42]. In 2017, Xie et al. proposed ResNeXT [235], which is an extension of ResNet and VGG, and achieved the state-of-the-art performance of 3.03% top-five errors. Around the same time, the problem of supervised image classification was regarded as “solved”, and the ImageNet classification challenge concluded. However, in many applications, the tasks cannot be formulated as plain vanilla image classification problems. Many object classes may be present in a single image. Therefore, more research efforts are being made toward object detection and segmentation.

#### 4.1.2. Object Detection

Object detection is also a fundamental problem in computer vision. Its aim is to identify and localize different objects in an image. Therefore, deep learning models for this problem usually consist of two components: The backbone component, which is similar to an image classification model, and the region proposal component, for predicting bounding boxes. Region-proposal and Region-based CNN (R-CNN) is a pioneering work for object detection [236]. However, it requires much computing time and memory for training. Therefore, several improved variants of R-CNN have been proposed, such as the renowned Fast R-CNN [55] and Faster R-CNN [44]. Another kind of models for object detection is represented by YOLO (You Only Look Once), which achieved reasonable performance for real-time object detection [46]. Other advanced models include Region-based Fully Convolutional Networks (R-FCNs) [237], which use ResNet as an object detector and are faster than the Faster R-CNN, and the Single-Shot MultiBox Detector (SSD) [238], which is even faster than YOLO and has comparable accuracy to Faster R-CNN. Most object detection methods mentioned above incur a high computational cost due to their bounding box processing. More architectures addressing this issue and achieving higher accuracy can be found in recent overview articles [239,240].

#### 4.1.3. Semantic Segmentation

Semantic segmentation refers to the process of dividing an image into meaningful types of regions. It is a vital step for many image processing and analysis tasks. Its aim is to label an image at the pixel level, or more accurately to assign each pixel to the class it most likely belongs to. A predominant and outstanding architecture particularly for the semantic segmentation problem is U-Net [43]. Due to the huge success of U-Net, many variants have been proposed to fit specific applications and achieved great results [241,242,243,244,245]. U-Net was originally proposed for addressing medical image segmentation. Similarly, other domain-oriented deep neural networks have been developed, including SegNet [246], PSPNet [247], and DeepLab [248]. More detailed discussions of semantic segmentation can be found in various surveys [249,250,251]. In a way, semantic segmentation can be seen as a rough classification, for example two different dog objects being segmented as one entity, but in many applications we need a finer segmentation, where each dog is segmented out separately. Hence, instance segmentation methods are important.

#### 4.1.4. Instance Segmentation

Instance segmentation is essential for many real-world application scenarios such as autonomous driving, medical imaging, robot vision, and so on. It refers to detecting instances of objects and, more challenging, demarcating their boundaries. Because instance segmentation involves both the detection of different objects and their per-pixel segmentation, networks for solving it can be either R-CNN driven or FCN driven. Mask R-CNN [47] is one of the representative networks. Its overall structure is the two-stage object detection network Faster R-CNN, where the box head is used for detection and the mask head for segmentation. Fundamentally, these instance segmentation networks employ object detection networks in identifying the object bounding boxes, while an extra component mask head is used to further extract the foreground of the bounding boxes. Other reputable networks include YOLACT [252] and SOLO [253] inspired by YOLO, and PolarMask [254] and AdaptIS [255] inspired by the object detection network FCOS [256]. See several recent reviews of instance segmentation for more details [257,258,259].

#### 4.1.5. Pose Estimation

Pose estimation refers to the problem of inferring the pose of a person or an object in an image or video. In other words, it concerns determining the position and orientation of the camera relative to a given person or object of interest. This is typically achieved by identifying, locating, and tracking a number of key points of the person or object. This problem is basic and important because it occurs in many real-world applications such as object/person tracking. Deep learning is often employed to detect and track these key points. There are many specific neural network architectures for this purpose, and the most robust and reliable ones that make good places to start include Stacked-Hourglass networks [260], Mask R-CNN, and PoseNet [261]. Key factors in designing DNNs for pose estimation include using dilation convolution, upsampling operations, and skip connections. This is because pose estimation requires a higher-resolution feature map and is more sensitive to the location of keypoints compared to classification/detection tasks. More advanced networks have been described in various reviews [262,263,264].

#### 4.1.6. Style Transfer

Style transfer refers to the computer vision task of blending two input images, named content image and style reference image, and producing an output image that maintains the core elements of the content image while following the style of the style reference image. It can power practical applications such as photo and video editing, gaming, virtual reality, and so on. Neural networks have become the state-of-the-art method for style transfer. Generally, CNNs are the mainstream approaches for this problem, and a style transfer model usually consists of two networks, namely a pretrained feature extraction network and a transfer network. Significant networks that are good starting points include the model proposed by Johnson et al. [265] for single style transfer, the model proposed by Dumoulin et al. [266] at Google for multiple style transfer, and the model proposed by Huang et al. [267] for arbitrary style transfer. Detailed explorations and more advanced architectures can be found in recent surveys [107,268,269].

#### 4.1.7. Video Analytics

Video analytics refers to generating descriptions of the content of, or events in the video, which involves tasks of object (persons, cars, or other objects) detection, tracking, as well as calculating their appearance and movements. It is also an important and essential computer vision technique and has significant practical benefits such as monitoring video for security incidents helps prevent crime, intelligent traffic systems, and more [270,271]. While its tasks overlap beyond image analysis tasks, they are more challenging because they involve both spatial and temporal information.

The object detection problem in video is associated with the object segmentation problem, because an accurate object segmentation facilitates object detection, and robust object detection in turn facilitates object segmentation. Recent neural networks for this problem are based on recurrent convolution neural networks (RCNN). For example, Donahue et al. [272] firstly proposed a long-term RCNN, where a set of CNNs is employed for visual understanding and then their outputs are fed to a set of RNNs for analyzing temporal information. Other representative RCNN based models include the one proposed by Ballas et al. [273], MaskRNN [274], and MoNet [275]. For comprehensive discussions of video analytics we refer to recent surveys [270,271,276,277].

#### 4.1.8. Codes, Pretrained Models, and Benchmark Datasets

Various implementation codes and pretrained models of many of the above introduced methods can be found in the references provided in Section 2.5.2. Some renowned benchmark datasets that are widely used in general computer vision to evaluate different deep learning methods are listed as follows.

(1) MNIST: http://yann.lecun.com/exdb/mnist/ (accessed on 2 November 2022).
(2) CIFAR-10 and CIFAR-100: https://www.cs.toronto.edu/kriz/cifar.html (accessed on 2 November 2022).
(3) ImageNet: https://image-net.org/challenges/LSVRC/ (accessed on 2 November 2022).
(4) COCO: https://cocodataset.org/#home (accessed on 2 November 2022).
(5) PASCAL VOC: http://host.robots.ox.ac.uk/pascal/VOC/ (accessed on 2 November 2022).
(6) OpenImages: https://storage.googleapis.com/openimages/web/index.html (accessed on 2 November 2022).
(7) MIT pedestrian: http://cbcl.mit.edu/software-datasets/PedestrianData.html (accessed on 2 November 2022).
(8) Youtube-8M: https://research.google.com/youtube8m/ (accessed on 2 November 2022).
(9) SVHN: http://ufldl.stanford.edu/housenumbers/ (accessed on 2 November 2022).
(10) Caltech: http://www.vision.caltech.edu/datasets/ (accessed on 2 November 2022).

### 4.2. Biomedical Sensor Systems

Deep learning has fundamentally converted the way we process, analyze, and interpret data, including in biology and medicine. We discuss deep learning in biomedical sensor systems from the perspective of three different biomedical domains: biomedical imaging, omics data analysis, and prognostics and healthcare.

#### 4.2.1. Biomedical Imaging

Biomedical image analysis is one of the most important and fundamental areas in biomedical science and has become a cornerstone of modern healthcare. Automatic biomedical image analysis involves a set of basic tasks introduced in Section 4.1, such as image reconstruction and registration, image or object classification, object detection, segmentation, and tracking. According to the different image types and their unique characteristics, we further divide biomedical imaging into four subareas and discuss the application of deep learning in each, as in [278].

(1) Medical Imaging. Medical images are typically acquired using devices such as X-ray CT (computed tomography), MRI (magnetic resonance imaging), and US (ultrasound). With the advancement of medical imaging devices, the quality of medical images has improved over the years, but their automated analysis is still a challenging task. DNNs can provide powerful solutions to this problem. For example, U-Net [43] and UNet++ [279] are two most reputable and popular architectures for medical image segmentation. In fact, U-Net has become the de facto standard method in medical image segmentation due to its huge success in the field. Various CNN-based architectures have achieved top performance for brain tumor analysis [280]. For a more in-depth discussion of DNN architectures in medical imaging, we refer to recent overview and survey papers [281,282,283].
(2) Pathological Imaging. Pathological images are generated from specimen slides by virtual microscopy, also called whole-slide imaging. Their visual interpretation is more challenging than for medical images due to the large size and high resolution of the images. As in medical imaging, deep learning brings great potential in providing reliable image interpretation in this subarea. For example, Zhu et al. [284] proposed a DeepConvSurv model based on CNN for survival analysis with pathological images. Li et al. [285] proposed a DenseNet based solution for pathological image classification. A recent trend in pathological image processing is to incorporate multiple instance learning to deal with the high resolution and weak labels of pathological images. More advanced models can be found in a recent survey paper [286].
(3) Preclinical Imaging. Preclinical imaging refers to the visualization of small living animals for conducting in-vivo studies for clinical translation. Preclinical images can be obtained by micro-US, MRI, and CT for anatomical imaging, or bioluminescence, PET (positron emission tomography), and SPECT (single photon emission computed tomography) for molecular visualization. Employing deep learning for interpreting these images is comparatively under-researched. A few related DNN-based methods are discussed in recent works [287,288].
(4) Biological Imaging. Biological images capture various aspects of organisms and biological systems that are not visible to the naked eye. Automated analysis and interpretation of these images is challenging, as they are typically very noisy and highly variable depending on experimental conditions, and they can be quite large. DNNs have proven to be very suitable for biological image analysis and have empowered biological research [289,290]. Moreover, to facilitate the design of DNN architectures for this purpose, neural architecture search-based solutions have been proposed for cell segmentation [291,292]. Architectures for deep learning-based biological image analysis have been discussed in several recent papers [293,294,295].

#### 4.2.2. Omics Data Analysis

Omics data are complex, heterogeneous, and high-dimensional, and deep learning methods are specially suitable to analyze them. According to the different types of data, we introduce deep learning in omics data analysis from the following aspects.

(1) Genomics. Deep learning methods have been applied to genomics data analysis for several years, and have achieved impressive results. For example, CNNs have been employed for single-nucleotide polymorphisms and indels detection [296]. SAEs have been successful in predicting the effect of genetic variants on gene expression [297]. Both have achieved better results than traditional methods. A review of more architectures can be found in a recent survey paper [298].
(2) Transcriptomics. Analysis of transcriptomics data may yield an estimate of the expression level of each gene or transcript across several samples [299]. Therefore, it can be seen as a typical deep learning problem. Various deep learning methods have been proposed for addressing this problem. For example, a RAN-based solution for detecting long ncRNAs achieved a remarkable 99% accuracy [300]. For comprehensive introductions and discussions we refer to various survey papers [301,302].
(3) Proteomics. Protein data analysis mainly centers around two topics: protein structure prediction (PSP) and protein interaction prediction (PIP) [303]. For PSP, deep learning-based methods have been used to solve problems such as backbone angles prediction [304], protein secondary structure prediction [305], and protein loop modeling and disorder prediction [306]. Moreover, due to the success of deep learning in generating higher-level representations and ignoring irrelevant input changes, deep learning methods have become the technology of choice to help PSP. For PIP, deep learning-based methods have been used to analyze protein–protein interactions [307], drug–target interactions [308], and compound–protein interactions [309]. A latest trend in PSP is using GNNs to better learn complex relationships among protein interaction networks for PSP.

#### 4.2.3. Prognostics and Healthcare

Clinical data and electronic medical records are vital for prognostics and healthcare management. Deep learning to handle these kinds of data is also rapidly growing [310,311]. For example, deep learning-based methods have been used for detecting cardiac arrhythmia from electrocardiograms [312] and for phenotype discovery using clinical data [313]. There are also examples of using DNNs and topic modeling techniques to learn effective representations from electronic health records [314,315]. A key challenge in this area is the efficient utilization of temporal information for achieving high performance [316]. Hybrid DNNs such as those incorporating RNN and CNN components are promising in addressing this challenge.

#### 4.2.4. Codes, Pretrained Models, and Benchmark Datasets

Various implementation codes and pretrained models of many of the above introduced methods can be found in the references provided in Section 2.5.2. In addition, the implementation of nnU-net [317], which is a powerful self-adapting neural network framework that can automatically configure itself, including selecting the optimal preprocessing, architecture, training, and post-processing for any new task, is publicly available (https://github.com/MIC-DKFZ/batchgenerators accessed on 2 November 2022). Some renowned benchmark datasets that are widely used in the biomedical domain to evaluate different deep learning methods are listed as follows.

(1) Decathlon: http://medicaldecathlon.com/ (accessed on 2 November 2022)
(2) MedPix: https://medpix.nlm.nih.gov/home (accessed on 2 November 2022)
(3) NIH Pancreas-CT: https://academictorrents.com/details (accessed on 2 November 2022)
(4) AMRG Cardiac Atlas: http://www.cardiacatlas.org/studies/amrg-cardiac-atlas/ (accessed on 2 November 2022)
(5) Cancer Imaging Archive: https://wiki.cancerimagingarchive.net (accessed on 2 November 2022)
(6) OASIS Brains: http://www.oasis-brains.org/ (accessed on 2 November 2022)
(7) ADNI: https://adni.loni.usc.edu/data-samples/access-data/ (accessed on 2 November 2022)
(8) DDSM: http://www.eng.usf.edu/cvprg/ (accessed on 2 November 2022)
(9) CTC: http://celltrackingchallenge.net/ (accessed on 2 November 2022)
(10) ISIC Archive: https://www.isic-archive.com/#!/onlyHeaderTop/gallery (accessed on 2 November 2022)

### 4.3. Biometric Sensor Systems

Biometrics deals with recognizing people by using their physical and behavioral characteristics. Biometric recognition can be formulated as a verification or identification problem. The verification task aims to verify whether a person is who they claim to be by comparing the person’s biometric template with the reference template of the claimed identity. The identification task compares a person’s biometric template with references of all identities in the database to establish the person’s identity. In either task, the system needs to collect the biometric data, extract features, and perform comparison or classification. Deep learning has a big impact on biometrics in terms of feature extraction and classification, which primarily involves supervised learning. Recent advances in this field also applied generative models with unsupervised learning to enhance the learning of features and improve recognition performance. In this section, we review deep learning approaches for biometric applications and discuss how the methods can benefit the field of biometrics and the open research questions.

#### 4.3.1. Automatic Face Recognition

Faces are one of the most commonly used biometrics in surveillance, forensics, security, access control applications scenarios. Acquisition of face biometrics is based on cameras and the collected data are in the format of images or videos. While being noninvasive and convenient, face biometrics are subject to imaging conditions and physical factors related to illumination, pose, expression, aging, and other appearance changes.

Conventional methods for automatic face recognition can be categorized into feature-based approaches and appearance-based approaches, which extract local features and global representations, respectively. With a hierarchical structure, deep learning simultaneously extracts local and global representations while handling nuisance factors. Among different architectures, CNN-based models show the most significant impact in this field. For example, CNNs with different architectures and loss functions [318] were trained to learn DeepID features in joint identification-verification tasks. Verification essentially deals with the similarity between two faces, and therefore, metric learning such as joint Bayesian and triplet loss are adopted. Identification, on the other hand, is a multiclass classification problem, hence the cross-entropy is usually used in the loss function. Facebook proposed DeepFace [319], which is a nine-layer CNN trained on four million Facebook images from four thousand subjects. DeepFace addresses the alignment issue and learns effective face representations with high transferability. Google proposed FaceNet, a deep CNN with triplet loss [320] to learn direct embeddings of images, which are effective in face verification, identification and clustering tasks. In addition, various CNN frameworks are proposed to handle the pose and illumination variations. For example, the face identity-preserving framework [321] integrates feature extraction layers with a reconstruction layer to reconstruct face images in a canonical view. An ensemble of pose-aware CNNs [322] was proposed for face recognition, where each model is trained for a specific pose using pose-specific images generated by 3D rendering.

To improve the efficiency of training deep neural networks, researchers have proposed different learning strategies. For example, a sparse network can be trained iteratively from a denser model using correlations between neural activations of consecutive layers [323]. A face alignment network [324] trained jointly with the face recognition network can reduce the number of training samples needed. Furthermore, hybrid discriminative and generative models were proposed to learn identity-specific representations that are pose-invariant [325]. Generative models such as AEs and GANs are also used to generate identity-bearing facial images [326]. In addition, the recognition of facial attributes such as age and gender [327] is an important task because it helps narrow down candidate matches, which can then facilitate face recognition. Hierarchical representations from a CNN or an ensemble of CNNs have been used for this purpose via classification [327] or regression [328]. CNN with different constructs have been adopted, including the residual network [329]. Training such models requires crowdsourcing to get the age and gender labels, which usually results in small datasets not sufficient to train deep neural networks. Therefore, models such as VGGNet and GoogleNet pretrained on large datasets such as ImageNet are often adopted and fined-tuned for age and gender estimation [330].

#### 4.3.2. Periocular Region and Iris

The periocular region presents salient traits for face and facial attribute recognition, which is helpful when the lower half of a face is occluded. Researchers have used CNN and RBM models trained with unsupervised learning to learn representations from periocular image patches and transferred the representations to recognition tasks [331]. Deep learning models were also used with conventional handcrafted methods to enhance performance. For example, autoencoders were used to learn latent representations from the texture features extracted by handcrafted filters [332], and CNNs were trained on both face images and the SIFT features to gain higher recognition accuracy [333].

The iris is a highly distinctive biometric trait. However, the acquisition of iris images often suffers low user acceptance. Iris recognition relies on random texture information in the irises and the quality of the extracted information depends on the preprocessing steps, including iris segmentation, off-axis gaze correction and removal of eyelashes. Gabor filtering is the classic method widely used in real-world applications for capturing iris texture information [334]. Deep learning replaces the Gabor filters with neural network modules. For example, CNNs were used to learn source-specific filters for iris images from visible and near-infrared sources [335]. Deep CNNs integrating inception layers were proposed for iris recognition, providing robust performance in terms of segmentation and alignment. Sparse autoencoders were also trained for feature extraction in mobile applications where iris images were collected by mobile devices [336]. Moreover, representations learned by CNNs were fused with handcrafted features to improve recognition accuracy.

#### 4.3.3. Fingerprint and Palmprint

Fingerprints are one of the most established biometric modalities. The acquisition of fingerprints uses cameras and the collected data are images. Two types of features are used, one is global features in terms of loop, delta, and whorl, and the other is local features in terms of ridge, valley, and minutiae. The major challenges of fingerprint recognition are the intra-subject variations caused by displacement, distortion, pressure, skin condition, and other noises. Applying deep learning to fingerprint recognition aims to extract deep global and local representations, as well as enhancing the fingerprint images.

CNNs are the most popular models in fingerprint biometric applications. With different designs in the neural network structures and training strategies, CNNs have been used for identification [337], authentication [338], liveness detection [339], double-identity detection [340], fingerprint alteration detection [341], spoofing detection [341], latent fingerprint recognition [342], cancellable recognition systems [343], and fingerprint segmentation [344], enhancement [345], and indexing [346]. Recent work also started to explore the use of CNNs for contactless and partial 3D fingerprint recognition [347,348,349,350,351]. DBNs are also used for fingerprint liveness detection, anti-spoofing, and enhancement [352]. One of the biggest challenges for most fingerprint recognition systems is the spoofing attack, which tries to circumvent a recognition system using artificial replicas of human characteristics similar to the legitimate enrolled trait. Models based on AEs such as stacked AEs and sparse AEs [353] were proposed to defend against spoofing attacks on fingerprint recognition systems and to perform liveness detection. Moreover, generative models based on GANs are widely used to generate fingerprint images [354]. The generation of high-quality fingerprints is used for fingerprint recovery [355] and presentation attack detection [356]. Furthermore, hybrid deep learning models or ensemble DL methods have been proposed to perform multiple tasks at once. For example, the Inception, MobileNet, and GAN are integrated in one framework [357] for localization and detection of altered fingerprints in order to address obfuscation presentation attack.

Palmprint and hand geometry share similar traits as fingerprints. Classic features include the hand/palm shape, principal lines, wrinkles, delta points, and minutiae features. Deep learning is used to learn these multiscale features from the palmprint and hand images. Various models based on CNN and RBM [358] have been used for palmprint recognition. The models are trained with either the whole palmprint images or regions of interest [359].

#### 4.3.4. Voice-Based Speaker Recognition

Application scenarios of speaker recognition can be classified into speaker verification, speaker identification, speaker diarization (which is used for automated speech transcription systems where dialogue is generated along with the speaker’s information), and speaker recognition in-the-wild (which refers to real-world scenarios where conditions are unknown or even corrupted with noise, echo and cross-talk). The in-the-world scenario is one of the major challenges targeted by researchers. The general types of speaker recognition are text-dependent and text-independent, and their difference lies in whether specific phrases are required or not. Deep learning methods are currently the state-of-the-art in the above-mentioned application scenarios and types. These methods process voice inputs in two patterns: raw sound waves and preprocessed data. Although some methods (e.g., the SincNet [360], RawNet [361], and AM-MobileNet [48]) are directly trained using raw speech data, most methods rely on signal preprocessing, which segments the signal into frames, performs normalization, converts signals to the frequency domain, and extracts spectrogram, mel-filterbank and mel-frequency cepstral coefficients (MFCC).

Based on the learning strategies, existing DL methods for speaker recognition can be categorized into stage-wise approaches and end-to-end systems. The stage-wise strategy involves two stages: speaker-specific feature extraction and classification of speakers. The i-vector [362] is a classic method for speaker recognition, consisting of a feature extractor based on Gaussian mixture models (GMM) and universal background models (UBM) and a classifier based on linear discriminant analysis. Inspired by i-vector, DL architectures are proposed to dig deeper representations, resulting in DL-based speaker embedding systems, d-vectors [363] (deep vector), x-vectors [364] (time-delay), and t-vectors [365] (triplet network). End-to-end systems do not require a multistage network. However, pretraining steps such as extraction of spectogram, MFCC, and mel-filterbank, or automatic feature learning with AEs, are employed to enhance recognition performance. Residual networks are widely used in feature extraction and end-to-end speaker recognition systems. Representative methods include the DeepSpeaker [192] (which integrates CNN with residual network), RawNet [361] (which consists of convolutional layers and gated recurrent unit layers with residual block constructs), and AM-MobileNet [48]. Some architectures adopted speech specific layers to facilitate speech signal processing. For example, the SincNet [360] uses a parameterized Sinc function to perform convolutions, which results in a smaller number of parameters and achieves better performance and faster convergence than standard CNNs. Autoencoders have also been widely used in speaker recognition for data encoding, feature dimension reduction, and data denoising [366]. Furthermore, generative models based on GANs are used for data augmentation and generation in speaker recognition systems to help extend short utterances into long speeches to enhance recognition performance [367]. An example is the SpeekerGAN [368] which is a variant of conditional GAN trained on inadequate speech data.

#### 4.3.5. Behavioral Biometrics

Handwritten signature is the most popular behavioural biometrics that has been widely used in various applications in legal, medical, and banking sectors. Based on how the signature is acquired, signature verification can be operated in two scenarios, including offline methods that use static signature images as inputs and online methods that further take into account the dynamics of the signing process (such as the pressure and velocity). Various deep learning models have been proposed to extract deep representations from the signature images and the signing process to improve verification accuracy for both offline and online applications. Popular models include the RNNs [369] (LSTM, gated recurrent unit), CNNs [370], DBN [371], and the combination of these models with AEs [372]. Classic methods such as the length normalized path signature descriptors [373], direction features, and wavelet features [371] are also used as inputs to train deep nets, instead of raw images, to improve performance. A Siamese network structure with contrastive loss was used for writer-independent verification [374], which measures how likely two given signatures are written by the same writer without knowledge of the writer’s identity.

Gait and keystrokes are two other popular behavioral biometrics which use the shape and motion cues of a person’s walking style and the typing patterns respectively for person recognition. There are two ways to acquire gait data: one is to use cameras or motion sensors to capture image/video [375] during the gait phases and the transition periods between phases, the second is to use sensors such as accelerators [376] to capture the signal variations of the person during walking. Deep learning methods for image/video-based gait recognition share great similarities with those in computer vision applications. The major difference lies in the input images, where models for gait recognition are usually silhouette shape-based and are trained with gait energy images [375] or chrono-gait images [377]. In terms of models, CNNs, LSTM, AEs, and their combinations are popular. In particular, 3D CNNs with temporal information in gait sequences considered provide a significant improvement in performance [378].

#### 4.3.6. Physiological Signals-Based Biometrics

Brain biometrics and heart biometrics are the major modalities in this category, which are an emerging branch of biometric technology. Brain biometrics are based on EEG (electroencephalogy) signals, which are recordings of the electrical pulses of the brain activity collected from a person’s scalp using electrodes. Similarly, the heart signals are collected from the chest, finger, or arm using electrical, optical, acoustic and mechanical sensors. The resultant signals are referred to as ECG (electrocardiography), PPG (photoplethysmography), PCG (phonocardiogram), and SCG (seismocardiogram), respectively. Other physiological signals used for biometric applications include the EMG (electromyography), EDA (electrodermal activity), and EOG (electrooculogram). Deep learning contributes to brain, heart, and other physiological signals-based biometrics in two aspects: automatic representation learning and classification. Various models based on MLPs, LSTM, and CNNs [379] are proposed to directly learn deep representations from the physiological signals for biometric recognition for end-to-end systems. In addition, since the salient features of these signals are usually in the frequency domain, pretraining steps such as Fourier transform and wavelet package decomposition were adopted in many works to convert the signal into the frequency or time-frequency domain [380]. Other pretraining steps include constructing functional connectivity networks using multichannel EEG signals, followed by CNNs [381] or GCNNs [382] to learn structural representations from the networks. The acquisition of physiological data from human subjects is a difficult and time-consuming task, and therefore, the datasets are usually small. To address this issue, generative models based on AEs [383,384] and GANs [385] were proposed for data augmentation and incomplete data reconstruction. The results show a significant improvement in recognition performance with data augmentation. We refer to [386] for a comprehensive survey in this area.

#### 4.3.7. Databases

Databases commonly used for biometric performance evaluation are summarized in Table 3. We separate the databases for different biometric modalities.

### 4.4. Remote Sensing Systems

In general, remote sensing refers to non-contact and long-distance detection technology, which uses remote sensors to capture the radiation and reflection characteristics of objects on the earth’s surface [391]. Remote sensors, typically mounted on airborne and satellite platforms, are the core component in any remote sensing system, and can be classified into two types: passive and active sensors. Passive sensors measure energy that is naturally available, and are usually optical and camera-based, such as panchromatic and multispectral sensors, providing images in the visible range. Different from passive sensors, active sensors such as radar sensors receive the reflection of the impulse they emitted and are less influenced by the environment.

With the availability of remote sensing imagery, DL methods have seen a rapid surge of interest from the remote sensing community and made a remarkable breakthrough. Deep learning in remote sensing confronts some new challenges:

(1) Multiple image modalities. Multimodality remotely sensed datasets, such as multi- and hyperspectral data, light detection and ranging (LiDAR) data, and synthetic aperture radar (SAR) data differ from each other not only in the imaging mechanism but also in the imaging geometries and contents. Different data modalities are often complementary. The design of deep models is crucial in making the most of these data.
(2) Growing importance of prior knowledge. Remote sensing data presents the real geodetic measurements for the earth surface, with each data point containing geophysical or biochemical information. Hence, minimizing distortion and improving data quality are especially crucial to remote sensing tasks. Pure data-driven models, without any prior knowledge, will lead to possible misinterpretation or blind trust.

In the following sections, we will investigate how deep learning models are modified to cope with these two challenges from the perspective of image classification, scene classification, object detection and segmentation, and multimodal data fusion.

#### 4.4.1. Image Classification

Image classification is one of the most active research topics in remote sensing, which aims to assign semantic labels to every pixel in the image. Various works used machine learning algorithms such as random forest (RF) and support vector machine (SVM) to improve the accuracy. The advent of deep learning pushed the boundary even further. Chen et al. [392] proposed the first deep learning-based classification model, which uses a stacked AE to extract hierarchical spectral information. Soon afterwards, DBN [393], and sparse SAEs [394] were introduced to learn stable and effective features for hyperspectral data classification. Makantasis et al. [395] proposed to use CNNs as feature extractor and a multiLayer perceptron (MLP) responsible for the classification task. Santara et al. [396] constructed an end-to-end CNN-based framework and generated band specific spectral-spatial features for classification. In [397], Li et al. proposed a pixel-pair strategy for CNN-based classification, and achieved state-of-the-art performance even with limited training samples. Recent improvements can be attributed to (1) specially designed architectures such as Siamese CNNs [398], the capsule network [399], and Transformer [400], and (2) improved feature representation [401,402].

#### 4.4.2. Scene Classification

Scene classification, which aims to automatically classify the image into the category it belongs to, has become one of the most active areas of high-resolution remote sensing image understanding, and attracted growing attention in the past decade [403]. It is a relatively challenging task because even different scenes may contain objects with similar features. Such variations make scene classification considerably difficult. Compared with traditional approaches based on bag-of-visual-words (BoVW), deep models have distinct advantages in learning more abstract and discriminative features, thereby providing much better classification accuracy. Hence, most of the recent works paid much attention to building a robust and informative scene representation. Using PTMs [404,405] is a popular technique in scene classification, as it is difficult and time-consuming to train a CNN model from scratch with a limited number of training samples. Fine-tuning [406] also helps the PTMs adapt to the specific task and learn oriented feature representation for remote sensing images. Another family of methods focuses on feature selection [407], features aggregation [402,408,409], and fusion [410,411,412]. For example, Lu et al. [409] proposed a supervised feature encoding module and a progressive aggregation strategy to make full use of intermediate features. To cope with large intra-class variance caused by large resolution variance and confusing information, Zhao et al. [412] proposed a multigranularity multilevel feature fusion branch to extract structural information and fine-grained features.

#### 4.4.3. Object Detection

With the rapid development of intelligent earth observation, automatic interpretation of remote sensing data has become increasingly important. Object detection in remote sensing aims to identify ground objects of interest such as vehicles, roads, buildings or airports from images and correctly classify them. In recent years, DL-based methods have been dominating this research area and made remarkable progress.

Preliminary work for object detection in remote sensing images [413,414,415] borrows the coarse-localization-fine-classification pipeline and CNN models from the computer vision community. Zhu et al. [416] introduced AlexNet CNN [37] to extract robust features, combined them with an image segmentation method for localization, and finally employed an SVM classifier for detection. Chen et al. [413] presented a hybrid DNN (HDNN) for vehicle detection, which used a DNN as feature extractor and a MLP as classifier. To further adapt CNN models to remote sensing object detection, researchers also take the rotation-invariant characteristic and context information into consideration. Cheng et al. [417] used and a newly proposed rotation-invariant layer to cope with object rotation variations. To cope with performance drop resulting from object appearance differences, Zhang et al. [418] proposed to use attention-modulated features as well as global and local contexts to detect objects from remote sensing images.

The advent of two-stage models such as RCNN [236] and faster RCNN [44], and one-stage methods such as YOLO series [46,419,420], made another leap in detection accuracy. By adapting two-stage models, most work focuses on improving the quality of region proposals [421,422,423]. More recently, advanced deep architectures such as Transformer[424] have also been introduced to advance the performance.

#### 4.4.4. Multimodal Data Fusion

Data fusion, as a fundamental task in the field of remote sensing, has been extensively studied for decades. With the availability of multimodal remote sensing data, data fusion techniques are expected to integrate complementary information and help boost the performance of downstream tasks. We briefly discuss two main topics in this area: (1) pansharpening, and (2) task-specific data fusion.

The goal of pansharpening is to integrate panchromatic (PAN) images and multispectral (MS) images, which are two types of optical remote sensing images with inevitable trade-off between spectral diversity and spatial resolution [425]. In general, PAN images provide high spatial resolution but contain limited spectral information, while MS images have much higher spectral resolution with less spatial details. The key point in pansharpening is that while ensuring the spatial increment, the detail injection implemented should preserve the unified spatial–spectral fidelity for fusion products [426]. The first DL-based pansharpening was proposed by Huang et al. [427], in which a modified sparse denoising autoencoder (MSDA) algorithm was used to learn the relationship between high-resolution (HR) and low-resolution (LR) image patches. Masi et al. [428] utilized a shallow CNN to upsample the intensity band after the intensity–hue–saturation (IHS) transform. As pansharpening aims to maximize the spatial injection and minimize spectral distortion, much effort has been devoted to making network architectures good at extracting spatial details while preserving spectral information [429,430]. To this end, Yuan et al. [431] proposed a multiscale and multidepth CNN to better fulfill spatial detail extraction and improve the fusion quality. Yang et al. [432] designed structural and spectral preservation modules and trained the network in the high-pass domain for more effective spatial injection. Zhang et al. [426] introduced saliency analysis as a measure to indicate the demand for spectral and spatial details, and treated them differently in the CNN based fusion process.

Unlike pansharpening aiming only at producing high-quality fusion products, task-specific data fusion usually leverages feature-level or decision-level fusion with specific downstream tasks such as land cover mapping and object detection in a unified framework [433]. A simple way of utilizing multimodal data for training a NN-based model is to concatenate them into an N-dimensional input. In [434], Lagrange et al. found that combining a digital surface model channel with RGB data in the training process can help retrieve some specific classes. For the image classification task, Hong et al. [433] designed an extraction Network (Ex-Net) and a fusion Network (Fu-Net) to learn from two different types of modality. Experiments on HS-LiDAR and MS-SAR data reveal the superiority of multimodal data fusion. Irwin et al. [435] combined SAR, optical imagery and airborne LiDAR data for surface water detection, in which a multilevel decision tree is developed to synthesize the results from a single data source.

#### 4.4.5. Codes, Pretrained Models, and Benchmark Datasets

To fulfill the demand of training deep learning-based models, a number of datasets are proposed by research groups in the earth observation community. Details of the publicly available datasets are shown in Table 4.

### 4.5. Intelligent Sensor Based Cybersecurity Systems

Cybersecurity is the practice of protecting critical systems and sensitive information from digital attacks, such as intrusion attacks and malware attacks. This section briefly reviews deep learning applications used in the detection of the four types of attacks: intrusion detection, malware detection, phishing detection, and spam detection.

#### 4.5.1. Intrusion Detection

Intrusion detection has become an essential task in the cybersecurity field. The objective of an intrusion detection system (IDS) is to distinguish malicious activities in network traffic and protect sensitive information. The following is a summary of the common attack types used in intrusion attacks.

(1) Denial-of-Service (DoS) attacks, such as botnet and smurf, aim to crash a machine or network service by flooding it with traffic, rendering it inaccessible to its users.
(2) Distributed DoS (DDoS) attacks aim to interrupt the regular traffic of a targeted network by flooding the target or its surrounding infrastructure with huge quantities of network traffic.
(3) User-to-Root (U2R) attacks attempt to get root access as a normal user by exploiting system weaknesses.
(4) Remote-to-Local (R2L) attacks are attempts by a remote system to obtain unauthorized access to the root.
(5) Password-based attacks attempt to obtain access to a system by attempting to guess or crack passwords.
(6) Injection attacks use well-designed instructions or queries to steal sensitive information or obtain unauthorized access to a system.

Deep learning-based techniques have demonstrated exceptional performance for intrusion detection in complicated, large-scale traffic conditions. For example, several recent methods [446,447,448] have introduced neural networks based on DBNs to achieve improved detection accuracy on the NSL-KDD dataset [449]. However, DBNs-based methods have the drawback that they are computationally unfeasible to train in an end-to-end supervised manner. AEs are widely used as a preprocessing step in intrusion detection, followed by the application of a deep learning classifier. For example, Abolhasanzadeh et al. [450] proposed an AE-based model with seven layers to extract compact and discriminant representations of the input data, and achieved high detection accuracy on the NSL-KDD dataset. In addition, several recent methods based on AEs have considered using stacked AEs for intrusion detection [451,452,453,454]. Vu et al. [455] proposed combining variational AEs [94] with several classifiers, such as naive Bayes, SVM, decision tree, and random forest classifiers for intrusion detection, and achieved good results on the NSL-KDD and UNSW-NB15 datasets. These AEs-based methods have the drawback of requiring an additional model to perform classification in additional to the AEs. To address this drawback, recent deep learning-based methods increasingly use CNNs for intrusion detection systems [456,457]. Especially, the LSTM networks have proven very useful because they have the strong ability to process data in intrusion detection that is often structured as sequences of features evolving over time. Several intrusion detection methods in the literature are based on LSTM networks [458]. Among these methods, the one proposed in [459] adopts a three-layer LSTM network, which achieves high detection accuracies on the ADFA-LD and UNM datasets. Similarly, the method proposed in [460] adopts a cascade of three LSTM network modules, which achieve an impressive intrusion detection accuracy by combining them through a voting mechanism. In addition, to take full advantage of LSTMs in processing time series data and CNNs in extracting spatial patterns, several recent methods consider combinations of LSTMs and CNNs for intrusion detection. For example, the method described in [461] uses both a CNN and a hybrid LSTM-CNN to perform the intrusion detection. The method developed in [462] uses a hybrid LSTM-CNN model based on the LeNet. GANs have been used for intrusion detection because their advantage of learning in an unsupervised manner is very suitable for learning the characteristics of data distributions in specific situations (e.g., under normal conditions) in the IDS context. For example, Schlegl et al. proposed a CNN-based GAN [463] to learn the characteristics of data captured under normal conditions, which is then used to detect anomalies by computing the distance between freshly captured data and normal data. In addition, Zenati et al. [464] proposed to further improve the computational efficiency of the GAN in [463] to achieve a faster detection.

#### 4.5.2. Malware Detection

Malware is a malicious software that is disseminated to compromise a system’s security, integrity, and functioning. The types of malware include viruses, worms, trojans, backdoors, spyware, botnets, and so on. Deep learning in this field is mainly concentrated on malware detection and analysis. The developed techniques can be generally classified into two categories: PC-based and Android-based malware detection.

(1) *PC-based malware detection.* Deep learning can be used to learn the language of malware through the executed instructions, and thus to help extract resilient features. To achieve this goal, Pascanu et al. [465] firstly proposed a method based on the Echo State Network (ESN) and RNN to classify malware samples. Later, David et al. [466] proposed a DeepSign to automatically generate malware signatures, which does not rely on any specific aspect of the malware. This model uses stacked denoising AE (SDAE) and creates an invariant compact representation of the general behavior of the malware. In 2017, Yousefi-Azar et al. [467] proposed a generative feature learning-based method for malware classification and achieved a network-based anomaly detection using AE. Recently, two GAN-based methods for malware detection have been proposed [468,469]. Specifically, in [468], Kim et al. adopted a transferred deep convolutional GAN (tDCGAN) to generate the fake malware and learn to distinguish it from the real one, which achieves robust zero-day malware detection. In [469], latent semantic controlling GAN (LSCGAN) is proposed to detect obfuscated malware, where features are first extracted using a VAE and then transferred to a generator to generate virtual data from a Gaussian distribution.
(2) *Android-based malware detection.* Malicious Android apps detection is vital and highly demanded by app markets. Deep learning models can automatically learn features without any human interference. The first investigation of applying deep learning to Android malware detection was Droid-Sec [470], which learns more than 200 features from both the static and dynamic analysis of Android apps for malware detection. Later, Hou et al. [471] proposed DroidDelver to deal with Android malware threats, which firstly categorizes the API calls of the Smali code into a block and then applies a DBN for newly unknown Android malware detection. Su et al. [472] proposed the DroidDeep for Android malware detection, which is also a DBN-based model. In 2017, CNN was firstly applied to Android malware detection context by McLaughlin et al. [473]. They used CNN to extract raw opcode sequences from disassembled code, with the purpose of removing the need to count the vast number of distinct n-grams. Later, Nix et al. [474] proposed a CNN-based framework for Android malware classification, which gets help from API-call sequences. Specifically, a pseudo-dynamic program analyzer is firstly used to generate a sequence of API calls along the program execution path. Then, the CNN learns sequential patterns for each location by performing convolution alongside the sequence and sliding the convolution window down the sequence. Recently, Jan et al. [475] employed a Deep Convolutional GAN (DCGAN) for investigating the dynamic behavior of Android applications.

#### 4.5.3. Phishing Detection

Phishing is a form of fraud in which the attacker tries to learn sensitive information such as login credentials or account information by sending emails or other communication messages. Therefore, phishing detection is a vital task in cybersecurity. Deep learning has also been researched to facilitate the solving of this task. For example, Zhang et al. [476] proposed to detect phishing email attacks by using a 3-layer FCN which consists of one input layer, one hidden layer, and one output layer. In addition, in this network, tanh and sigmoid activation functions are used to better fit the data. Mohammad et al. [477] proposed a self-structuring neural network for detecting phishing website attacks. It can automate the process of structuring the network, which is important for extracting the dynamic phishing-related features. Benavides et al. [478] investigated a variety of networks for cyber-attacks classification and found that the most regularly utilized are DNN and CNN. Although diverse deep learning-based methods have been presented and analyzed, there is still a research gap in the application of deep learning in cyber-attacks recognition.

#### 4.5.4. Spam Detection

The research of spam detection can be basically classed into text spam detection and multimedia spam detection.

(1) *Text Spam Detection.* Text-based spam content generally includes malicious URLs, hashtags, fake reviews/comments, posts, SMS, chat messages, etc. Wu et al. [479] developed a deep learning-based method to identify spam on Twitter, which employs MLP classifiers to learn the syntax of many tweets to perform pre-processing and create high-dimensional vectors. It outperforms the traditional feature-based machine learning methods such as random forest. Jain et al. [480] proposed a semantic CNN (SCNN) that employs a CNN with an additional semantic layer for malicious URL detection, where the semantic layer is a Word2Vec network used to map the word. Thejas et al. [481] proposed a hybrid deep network for click fraud detection, which involves an ANN and auto-encoders (AEs). The ANN is used to gain learning and pass knowledge to the other layers in the hybrid neural network, while the AEs are used to acquire the distribution of human clicks. The proposed hybrid network achieved high accuracy on a real-time dataset of ad-clicks data. Singh et al. [482] proposed using a CNN to classify the aggressive behavior on social networks, which achieved significant accuracy. Ban et al. [483] proposed using a Bi-LSTM network to extract features from Twitter text for spam detection.
(2) *Multimedia Spam Detection.* Deepfake is a currently famous technology that synthesizes media to create falsified content by replacing or synthesizing faces, speech, and manipulating emotions. It uses deep neural networks to learn from large and real samples to simulate human behavior, voices, expressions, variations, etc., and thus, its generated content seems genuine [484]. This technology can be valued in many applications such as movies, games, education, etc. However, it can seriously eradicate trust due to giving forged reality [485]. It also brings many challenges for the spam detection, as its synthetic media is generated by deep learning techniques. Therefore, an arms race between Deepfake techniques and spam detection algorithms has begun. For example, Hasan et al. [485] proposed employing a blockchain-based Ethereum smart contract framework to deal with media content authenticity. This system can preserve all historical information related to the creator and publisher of the digital media, and then it checks the authenticity of video content by tracking whether it is from some reliable or trustworthy source or not. Fagni et al. [486] proposed a TweepFake to detect deepfake tweets, which involves CNN and bidirectional gate recurrent unit (GRU). For more advanced neural networks for multimedia spam detection we refer to the survey paper [487].

#### 4.5.5. Codes, Pretrained Models, and Benchmark Datasets

Various implementation codes and pretrained models of many of the above introduced methods can be found in the references provided in Section 2.5.2. The popular benchmark datasets for intrusion detection are summarized in Table 5.

### 4.6. Internet of Things (IoT) Systems

With the development of commodity sensors and increasingly powerful embedded systems, the research of IoT is rapidly emerging and developing. According to the different sensor systems in the IoT, we describe deep learning in this domain from four aspects: smart healthcare, smart home, smart transportation, and smart industry.

#### 4.6.1. Smart Healthcare

Deep learning and IoT in smart healthcare systems can be researched in the following two aspects.

(1) *Health Monitoring.* Sensor-equipped mobile phones and wearable sensors enable a number of mobile applications for health monitoring. In these applications, human activity recognition is used to analyze health conditions [493]. However, extracting effective representative features from the massive raw health-related data to recognize human activity is one of the significant challenges. Deep learning is employed for this purpose in these applications. For example, Hammerla et al. [494] proposed to use CNNs and LSTM to analyze the movement data and then combine the analysis results to make a better freezing gaits prediction for Parkinson disease patients. Zhu et al. [495] proposed using a CNN model to predict energy expenditure from triaxial accelerometers and heart rate sensors, and achieved promising results to relieve chronic diseases. Hannun et al. [496] proposed using a CNN with 34 layers to map from a sequence of ECG records obtained by a single-lead wearable monitor to a sequence of rhythm classes, and achieved higher performance than that of board certified cardiologists in detecting heart arrhythmias. Gao et al. [497] proposed a novel recurrent 3D convolutional neural network (R3D), which can extract efficient and discriminating spatial-temporal features for action recognition through aggregating the R3D entries to serve as an input to the LSTM architecture. Therefore, with wearable devices, it can monitor health state and standardize the way of life at any time. Deploying deep learning-based methods on low-power wearable devices can be very challenging because of the limited resources of the wearable devices. Therefore, some research works employing deep learning for health monitoring focus on addressing this issue. For example, Ravi et al. [498] utilized a spectral domain preprocessing for the data input to the deep learning framework to optimize the real-time on-node computation in resource-limited devices.
(2) *Disease Analysis.* Using the comparatively cheap and convenient mobile phone-based or wearable sensors for disease analysis is increasingly important for healthcare. Deep learning has been widely used in assisting this. For example, CNNs have been used to automatically segment cartilage and predict the risk of osteoarthritis by inferring hierarchical representations of low-field knee magnetic resonance imaging (MRI) scans [499]. Another work using CNNs is to identify diabetic retinopathy from retinal fundus photographs [500], which has achieved both high sensitivity and specificity over about 10,000 test images with respect to certified ophthalmologist annotations. Other examples of employing deep learning for disease analysis include the work of Zeng et al. [501], where a deep learning-based pill image recognition model is proposed to identify unknown prescription pills using mobile phones. In addition, Lopez et al. [502] proposed a deep learning-based method to classify whether a dermotropic image contains a malignant or benign skin lesion. Chen et al. [503] proposed a ubiquitous healthcare framework UbeHealth for addressing the challenges in terms of network latency, bandwidth, and reliability. Chang et al. [504] proposed a deep learning-based intelligent medicine recognition system ST-MedBox, which can help chronic patients take multiple medications correctly and avoid taking wrong medications.

#### 4.6.2. Smart Home

Smart home enables the interconnection of smart home devices through home networking for better living. In recent years, a variety of systems has been developed with the application of deep learning techniques. Two main kinds of smart home applications are indoor localization and home robotics, described below.

(1) *Indoor Localization*. With the spread of mobile phones, indoor localization has become a critical research topic because it is not feasible to employ Global Positioning System (GPS) in an indoor environment. Indoor localization covers several tasks such as baby monitoring and intruder detection. However, there are a lot of challenges to achieve these task, e.g, the multi-path effect, the delay distortion, etc. In addition, high processing speed and accuracy are essential for indoor localization systems. Fingerprinting-based indoor localization is a powerful strategy to address these challenges. For example, Gu et al. [505] proposed a semisupervised deep extreme learning machine (SDELM), which takes advantage of semi-supervised learning, deep learning, and extreme learning machine, and achieves a satisfactory localization performance while reducing the calibration effort. Mohammadi et al. [506] proposed a semisupervised DRL model, which uses VAEs as the inference engine to generalize the optimal policies. Wang et al. [507] proposed using an RBM with four layers to process the raw CSI data to obtain the locations. One challenge of applying deep learning in this field is the lack of suitable databases for large indoor structures such as airports, shopping malls, and convention centers. In addition, DRL-based fingerprinting is another area that has not received much attention. However, DRL is gaining enormous momentum and may push the boundaries of performance.
(2) *Home Robotics*. Equipped with commodity sensors, home robots can perform a variety of tasks in home environments. For example, popular tasks include localization, navigation, map building, human–robot interaction, object recognition, and object handling. However, case-specific strategies are needed for guiding a mobile robot to any desired locations when GPS is not available. In [508], a deep learning-based method for autonomous navigation to identify markers or objects from images and videos is proposed, which uses pattern recognition and CNNs. Levine et al. [509] proposed to train a large CNN to achieve successful grasps of the robot gripper using only monocular camera images. This method can predict the probability of the task-space motion of the gripper, and is independent of the camera calibration or the current robot pose. Therefore, it greatly improves the hand-eye coordination of a robot for object handling, and thus improve human–robot interaction. Reinforcement learning and unsupervised learning will be promising in this area because it is inefficient to manually label data that may change dramatically depending on the user and environment in a smart home.

#### 4.6.3. Smart Transportation

Nowadays, intelligent transportation systems heavily depend on the historical and real-time traffic data collected from all kinds of sensors, such as inductive loops, cameras, crowd-sourcing, and social media. Deep learning in various smart transportation systems currently has the following focuses.

(1) *Traffic Flow Prediction.* Traffic flow prediction is a basic and essential problem for transportation modeling and management in intelligent transportation systems. Deep learning has been increasingly used in this area to exploit the rich amount of traffic data and thus extract highly representative features. For example, Huang et al. [510] proposed using a DBN to capture effective features from each part of road traffic networks, and then these features from related roads and stations are grouped to explore the nature of the whole road traffic network to predict traffic flow. Lv et al. [511] proposed a stack of AEs model to extract features from historical traffic data to make the prediction. In addition, there are a lot of works focused on using deep learning for traffic and crowd flow prediction [512,513]. Most current methods to predict traffic flow are for short-term prediction while long-term prediction horizons can reduce costs and provide better intelligent transportation system management. Research in this field is very challenging due to the difficulty of achieving high accuracy of long-term prediction. A promising solution is using data-driven methods.
(2) *Traffic Monitoring.* Traffic monitoring is one of the most popular research fields in smart transportation. Its aim is to both reduce the workload of human operators and warn drivers of dangerous situations. Therefore, traffic video analytics is a key part of traffic monitoring. One of the key tasks in traffic monitoring is object detection, which includes pedestrian detection, on-road vehicle detection, unattended object detection, and so on. As in other tasks (Section 4.1), deep neural networks for object detection have also played an important role here, and have significantly improved the accuracy and speed of traffic monitoring. For example, Ren et al. [44] proposed using a region proposal network (RPN), which shares full-image convolutional features with the detection network and can achieve nearly cost-free region proposals. Redmon et al. [46] proposed to formulate frame object detection as a regression problem, which separates the processes of recognizing bounding boxes and computing class probabilities. Another important task in traffic monitoring is object tracking, which plays a significant role in surveillance systems, including tracking suspected people or target vehicles for safety monitoring, urban flow management, and autonomous driving. Deep learning has also been widely in this area. For example, Vincent et al. [451] proposed building deep networks based on stacking layers of denoising AEs for this purpose. Li et al. [514] proposed a robust tracking algorithm based on a single CNN to learn effective feature representations for the target object. Ondruska et al. [515] proposed an end-to-end object tracking approach, which uses RNN to directly map from raw sensor input to object tracks in sensor space.
(3) *Autonomous Driving.* Autonomous driving is crucial to city automation. Vision-based autonomous driving systems have two main paradigms: mediated perception-based and behavior reflex-based. The underlying idea of mediated perception-based methods is to recognize multiple driving-relevant objects, such as lanes, traffic signs, traffic lights, cars, and pedestrians. However, most of these systems rely on highly precise instruments and thus bring unnecessarily high complexity and cost. Therefore, current autonomous driving systems focus more on real-time inference speed, small model size, and energy efficiency [516]. Deep learning is adopted here to learn a map from input images/videos to driving behaviors, or to construct a direct map from the sensory input to a driving action. For example, Bojarski et al. [517] trained a CNN to map raw pixels from a single front-facing camera directly to steering commands. Xu et al. [518] proposed using an end-to-end FCN-LSTM network to predict multimodal discrete and continuous driving behaviors. Readers interested in finding more deep learning-based methods for this topic are referred to the survey paper [519]. Currently, most papers on deep learning for self-driving cars focus on perception and end-to-end learning. Although deep learning has made great progress in the accuracy of object detection and recognition, the level of recognition detail still needs to be improved to perceive and track more objects in real time in the autonomous driving scene. In addition, the gap between image-based and 3D-based perception needs to be filled.

#### 4.6.4. Smart Industry

Smart industry, also known as industry 4.0, represents the latest trend of the manufacturing revolution. In the era of smart industry, explosive data produced in manufacture can be analyzed by deep learning to empower the manipulators with human-like abilities. Deep learning in several main research topics are described as follows.

(1) *Manufacture Inspection.* Manufacture inspection refers to inspecting and assessing the quality of products. Various deep learning-based visual inspection methods have been proposed and become a powerful tool to extract representative features and thus to detect product defects in large scale production. For example, Li et al. [520] proposed a CNN-based classification model to implement a robust inspection system, which significantly improves the efficiency. Park et al. [521] proposed a generic CNN-based method to extract patch features and predict defect areas through thresholding and segmenting for surface integration inspection. Deep learning based methods have achieved the best experimental results so far in this domain, with accuracies ranging from 86.20% up to 99.00%.
(2) *Fault Assessment.* Fault assessment is crucial to building smart factories. Specific application tasks include machinery conditions monitoring, incipient defects identification, root cause of failures diagnosis, fault detection of rotating machines with vibration sensors, bearing diagnosis, tool wear diagnosis, and so on. This information can then be incorporated into manufacturing production and control. Deep learning has also been used here to solve these tasks. For example, Cinar [522] proposed using transfer learning models for equipment condition monitoring. Chen et al. [523] investigated the latest deep learning based methods for machinery fault diagnostics. Wang et al. [524] proposed a wavelet-based CNN to achieve automatic machinery fault diagnosis. Specifically, a wavelet transform is used to transfer a one-dimensional vibration signal into a two-dimensional one which is then fed into the CNN model. Wang et al. [525] proposed a continuous sparse auto-encoder (CSAE), which incorporates a Gaussian stochastic unit into its activation function to extract nonlinear features of the input data. Lei et al. [526] proposed a sparse filtering based two-layer neural network model, which is used to learn representative features from the mechanical vibration signals in an unsupervised manner. Generally, AE fits well with high-dimensional data and thus is a good technique of choice for fault assessment.
(3) Others. Deep learning has also been used in many sectors of renewable power systems. For example, Alassery et al. [527] proposed using neural networks for solar radiation prophesy models for green energy utilization in the energy management system. Another promising application of deep learning in the smart industry field is smart agriculture. For example, Khan et al. [528] proposed an optimized smart irrigation system for effective energy management, which overcomes the problems of transmitting data failure, energy consumption, and network lifetime reduction in the field of IoT-based agriculture. DNNs have also been applied in waste management. For example, Kshirsagar et al. [529] proposed using a customized LeNet model to classify garbage into cartons and plastics.

#### 4.6.5. Codes, Pretrained Models, and Benchmark Datasets

Implementation codes and pretrained models of many of the above introduced applications can be found in the references provided in Section 2.5.2. In addition, some commonly used datasets suitable for building deep learning applications in IoT are listed as follows.

(1) CGIAR Dataset: http://www.ccafs-climate.org/ (accessed on 2 November 2022)
(2) Educational Process Mining: https://archive.ics.uci.edu/ml/datasets/mining (accessed on 2 November 2022)
(3) Commercial Building Energy Dataset: https://combed.github.io/ (accessed on 2 November 2022)
(4) Electric Power Consumption: https://archive.ics.uci.edu/ml/datasets/power (accessed on 2 November 2022)
(5) AMPds Dataset: http://ampds.org/ (accessed on 2 November 2022)
(6) Uk-dale Dataset: https://jack-kelly.com/data/ (accessed on 2 November 2022)
(7) PhysioBank Databases: https://physionet.org/data/ (accessed on 2 November 2022)
(8) T-LESS: http://cmp.felk.cvut.cz/t-less/ (accessed on 2 November 2022)
(9) Malaga Datasets: http://datosabiertos.malaga.eu/dataset (accessed on 2 November 2022)
(10) ARAS Human Activity Datasets: https://www.cmpe.boun.edu.tr/aras/ (accessed on 2 November 2022)

### 4.7. Natural Language Processing (NLP)

NLP is a crucial and widely researched field. It is a subfield of AI that is concerned with enabling computers to understand text and spoken language in much the same way humans do. Due to the ambiguities of human language, NLP is a very challenging problem. Some involved popular tasks include speech recognition, sentiment analysis, machine translation, and question answering, introduced in the following.

#### 4.7.1. Speech Recognition

Speech recognition, also called speech-to-text, refers to the task of enabling a computer to translate human speech into text. There are many algorithms for speech recognition, but deep learning provides more advanced solutions. This is because DNNs can combine several aspects of the voice signals such as grammar, syntax, structure, and composition to understand and process human speech. The initial success in speech recognition was achieved by Zweig et al. [530] on a small-scale dataset with an error rate of 34.8%. After that, more advanced neural networks were proposed to improve recognition accuracy such as the representative networks Segmental RNN, EdgeRNN, and Quanaum CNN [531,532,533]. Comprehensive introductions of architectures for speech recognition can be found in recent survey papers [534,535,536].

#### 4.7.2. Sentiment Analysis

Sentiment analysis refers to the task of determining the attitude of reviewers, more specifically, the task of determining whether data are positive, negative, or neutral. It focuses on the polarity of a text but also aims to detect specific feelings and emotions such as happy and sad, and intentions such as interested and not interested. Popular types of sentiment analysis include graded sentiment analysis, emotion detection, and multilingual sentiment analysis. When applying deep learning to sentiment analysis, it is usually formulated as a classification problem, where DNN takes texts as input and outputs a category representing the sentiment class. For example, the Bag-of-Words (BoW) model is one of the most reputable methods for document level sentiment classification [537]. The Recursive AE (RAE) network proposed by Socher et al. is the first model for sentence level sentiment classification [538]. The Adaptive RNN (AdaRNN) is a renowned model for aspect-level sentiment classification [539]. More introductions and discussions of DNNs for sentiment analysis are given in other review papers [540,541].

#### 4.7.3. Machine Translation

Machine translation refers to the process of automatically translating text from one language to another without human involvement. This is one of the first applications of computing power, starting in the 1950s. Deep learning is well suited for this problem because DNNs can consider the whole input sentence at each step for generating the output sentence. This way, it can address the limitations of traditional methods that need to break an input sentence into words and phrases, and thus provide better translation quality. Basically, DNNs for machine translation have an encoder-decoder structure, where the encoder learns to extract the important features from its input sentence, and the decoder processes the extracted features and outputs the target sentence. For example, Kalchbrenner et al. [542] proposed a model with a CNN encoder and RNN decoder, which is the most original and classic structure of machine translation. More advanced architectures for machine translation are discussed in recent survey papers [543,544].

#### 4.7.4. Question Answering

Question answering refers to building systems that can answer questions posed in a natural language by humans. For this problem, a DNN takes a specific question and a paragraph of text as input and aims to output an answer to this question based on the given text. Such DNNs need to understand the structure of the language and have a semantic understanding of the context and the question, thus, attention-based DNNs are needed to handle the complex training. More specifially, attention-based RNNs are suitable for this task. One of the most famous networks for question answering is R-Net [545], which employs a gated attention-based RNN. Other renowned architectures include FusionNet [546] and the recently emerging Transformer. For comprehensive introductions and discussions on question answering we refer to recent surveys [547,548].

#### 4.7.5. Codes, Pretrained Models, and Benchmark Datasets

In addition to the references provided in Section 2.5.2, we refer to a survey of pretrained models for NLP [549]. A collection of renowned benchmark datasets that are widely used in NLP to evaluate different deep learning methods can be found at https://github.com/niderhoff/nlp-datasets/blob/master/README.md accessed on 2 November 2022. In addition, we list the most advanced pretrained language models as below.

(1) BERT: https://github.com/google-research/bert (accessed on 2 November 2022)
(2) GPT2: https://github.com/openai/gpt-2 (accessed on 2 November 2022)
(3) XLNet: https://github.com/zihangdai/xlnet (accessed on 2 November 2022)
(4) RoBERTa: https://github.com/facebookresearch/roberta (accessed on 2 November 2022)
(5) ALBERT: https://github.com/google-research/albert (accessed on 2 November 2022)
(6) T5: https://github.com/google-research/T5 (accessed on 2 November 2022)
(7) GPT3: https://github.com/openai/gpt-3 (accessed on 2 November 2022)
(8) ELECTRA: https://github.com/google-research/electra (accessed on 2 November 2022)
(9) DeBERTa: https://github.com/microsoft/DeBERTa (accessed on 2 November 2022)
(10) PaLM: https://github.com/lucidrains/PaLM-pytorch (accessed on 2 November 2022)

### 4.8. Audio Signal Processing

Audio signal processing was an early application of deep learning and is still one of its major application domains. Before deep learning, conventional methods for audio signal processing relied on handcrafted feature extraction, including the mel frequency cepstral coefficients (MFCCs), discrete cosine transform, and mel filter bank. Deep learning improves the processing performance by learning hierarchical representations from the audio signal using various models such as CNNs, RNNs, and GANs. These models are either trained using raw audio signals or classical features extracted from audio signals. This section briefly reviews the application of deep learning in the main scenarios of audio signal processing, including speech recognition, music and environmental sound analysis, localization and tracking, source separation, audio enhancement, and synthesis.

#### 4.8.1. Speech Recognition

Different from the speech recognition in Section 4.7.1, here speech recognition refers to converting speech into sequences of words in the context, which is the base for any speech-based interaction system. It is widely used in virtual assistance systems such as Google Home, Apple Siri, and Microsoft Cortana, and speech transcriptions such as the YouTube caption function. For a long time, the modeling of speech was dominated by methods based on Gaussian mixture models and hidden Markov models due to their mathematical elegance. However, deep learning models dramatically reduced the word error rate on various recognition tasks, and hence became mainstream [550]. Popular models for speech recognition include LSTMs, GRUs [551], and a combination of LSTM layers with convolutional layers [552]. RNN blocks (including LSTM and GRU) are widely used to model the temporal correlations in speech sequences. Sequence-to-sequence models such as CTC (connectionist temporal classification) [553] and LAS (listen, attend and spell) [554] were also proposed. Transfer learning also plays an important role to enhance systems on low resource language with data from rich resources languages [555]. In addition to speech recognition, other applications related to speech are voice activity detection, speaker recognition (see Section 4.3.4), speech translation, and language detection [556].

#### 4.8.2. Music and Environmental Sound Analysis

Music analysis involves low-level tasks such as onset/offset detection, fundamental frequency estimation, rhythm analysis, and harmonic analysis, and high-level tasks such as instrument detection, separation, transcription, segmentation, artist recognition, genre classification, discovery of repeated themes, music similarity estimation, and score alignment. These tasks were previously done by handcrafted features and conventional classifiers, and are now addressed by deep learning algorithms such as LSTMs, CNNs, and RNNs [557]. Modern systems integrate temporal modeling [558], applying 2D convolution on spectro-temporal inputs before doing 1D convolution to fuse representations across frequencies, followed by GRU to capture the sequence dependencies.

Environmental sound analysis is often used in multimedia indexing and retrieval, acoustic surveillance, and context-aware devices. In terms of recognition tasks, deep learning models are mainly used for acoustic scene classification [559] (the scene labels can be home and street), acoustic event detection [560] (detect the start and end time of an event and assign a label to the event) and tagging [561] (predict multiple sound classes at the same time).

#### 4.8.3. Source Separation, Enhancement, Localization and Tracking

Source separation aims to recover one or several source signals from a given mixture signal. It is an important task in audio signal processing in real-world environments, and is often performed before speech recognition to improve the data quality. In single-channel setups (only one microphone is used), deep learning aims to model the single-channel spectrum or the separation mask of a target source [562]. Convolutional and recurrent layers are often used in such models. Furthermore, some methods integrate supervised learning and supervised learning for source separation. For example, deep clustering [563] performs supervised learning to estimate embedding vectors for each time-frequency point, then cluster them in an unsupervised manner for separation. In multi-channel setups (e.g., audio data are collected from multiple microphones), the separation can be improved by taking into account the spatial locations of sources or the mixing process. In this case, the input of DNNs contains spatial features as well as spectral features, and the DNNs are used to estimate the weights of a multi-channel mask [564].

Audio enhancement aims to reduce noise and improve the audio quality. It is a critical component for robust systems. Deep learning in audio enhancement is mainly designed for reconstructing clean speech [565] or estimating masks [566] from noisy signals. To this end, researchers have proposed various models based on GANs [112], denoising AEs [567], CNNs [567] and RNNs [568].

For localization and tracking, deep neural networks are often trained on the phase spectrum [569], magnitude spectrum [570], and cross-correlation between channels [567]. The key is to design an architecture, e.g., a CNN, in a way that can learn the inter-channel information while extracting within-channel representations.

#### 4.8.4. Sound Synthesis

Sound synthesis can be used to generate realistic sound samples, speeches [571], music and art [572]. It is achieved by generative models which learn the characteristics of sound from a database and output desired sound samples. When the deep learning model is operated to generate fake speeches for a given person, it is often referred to as DeepFake. Popular deep learning models used for sound synthesis include VAEs and GANs [573], where the sound is synthesized and upsampled from a low-dimensional latent representation. Autoregressive approaches such as LSTM and GRU, on the other hand, generate new samples iteratively based on previous samples [574]. With multiple stacked layers, such methods are able to process sound at different temporal resolutions. The WaveNet [575] is a popular model in this regard. It stacks dilated convolutional layers, providing context windows of reasonable size to allow the model to learn context information (e.g., speaker identity). Furthermore, the problem of autoregressive sample prediction is cast into a classification problem. Follow-up models such as the parallel WaveNet [576] further improve the computational efficiency during the training stage.

### 4.9. Robotic Systems

Applications of deep learning in robotics are mainly aimed at addressing the challenges in learning complex and high-dimensional dynamics, learning control policies in dynamic environments, advanced motion manipulation, object recognition and localization, human action interpretation and prediction, sensor fusion, and task planning. In terms of deep learning architectures and strategies, existing methods for robotics can be classified into discriminative models, generative and unsupervised models, recurrent models, and policy learning models trained with reinforcement learning. This section briefly reviews how these models are used in different tasks.

#### 4.9.1. Learning Complex Dynamics and Control Policies

Robots often need to cope with states with high-level uncertainty, which requires the system to be able to quickly and autonomously adapt to new dynamics. This is important in tasks such as grasping new objects, traveling over surfaces with unknown or uncertain properties, managing interactions with a new tool or environment, and adapting to system degradation. Discriminative models, such as CNNs, were trained to assess the possibility of a specific robot motion for successfully grasping common office objects from image data [509]. DeepMPC [577] is a recurrent conditional deep predictive dynamics model for robotic food-cutting which is a controlling task with complex nonlinear dynamics. Transforming recurrent units were adopted to handle the time-dependent dynamics by integrating long-term information while allowing transitions in dynamics. Generative models such as AEs and GANs were also used to model the nonlinear dynamics of simple physical systems [578] and inverse dynamics of a manipulator [579]. Reinforcement learning plays a significant role in robotic control tasks. It is useful in learning to operate dynamic systems from partial state information. For example, it has been used to learn deep control policies for autonomous aerial vehicles control [580].

#### 4.9.2. Motion Manipulation

It remains elusive to find robust solutions for robotic motion tasks such as grasping deformable or complex geometries, using tools, and actuating in dynamic environments. The corresponding challenges approached with deep learning methods are grasp detection, path and trajectory planning, and motion control. Deep learning models based on recurrent units, CNNs [581,582], and deep spatial AEs [583] have been used for learning visuomotor and manipulation action plans.

#### 4.9.3. Scene/Object Recognition and Localization

Scene and object recognition as well as localization are critical tasks for robot systems, since knowing what kind of objects are there in the environment and the locations of those objects is a prerequisite for performing other tasks. Deep learning methods have shown promising performance in recognizing and classifying objects for grasp detection [581,584], including advanced applications such as recognizing deformable objects and estimating their state and pose for grasping [585], semantic tasks [586], and path specification [587].

#### 4.9.4. Human Action Interpretation and Prediction

Effective human–robot interaction requires the robot to have social skills, hence, the robot needs to be capable of inferring the intentions of humans and giving corresponding responses or actions accordingly. Such skills are critical in human–robot collaborative applications such as social robots, manufacturing, and autonomous vehicles. Interpreting and predicting human social behavior is a complex task, and it is difficult to formulate handcrafted solutions. Deep learning methods present great potential in this area. Learning by demonstration [581] is one way to solve the problem, where deep learning models are trained to learn manipulation action plans by watching unconstrained videos from the World Wide Web. In another study, a recurrent model was trained for the robot to learn grasping actions from a human collaborator [588].

#### 4.9.5. Sensor Fusion

The use of multiple sources of information is necessary in robotic systems, as it provides a plethora of rich representations of the environment and brings proper redundancy to the system to deal with uncertainties. The challenge is how to construct meaningful and useful representations from the various data sources. Due to the hierarchical structures, deep learning models naturally support the processing and integration of high-level representations learned from different data streams. For example, generative models [589] and recurrent models [590] with unsupervised learning were proposed for integrating multi-modal sensorimotor data, including video, audio, and joint angles, for robotic systems. The level of abstraction depends on the application specifics.

#### 4.9.6. Knowledge Adaptation in Robotic Systems

Training deep learning models can be time-consuming and data demanding. A robotic system should be crafted in a way that is easy to adapt to a similar task. Transfer learning plays an important role in leveraging the knowledge gained by previous solutions of similar problems to solve new problems. To this end, researchers have used pretrained models [236] and proposed sim-to-real approaches [591] to facilitate the learning process and improve efficiency. For example, AlexNet, GoogleNet, and VGG models pretrained on the ImageNet dataset have been used for extracting high-level representations from image data for object recognition in robotic systems. The sim-to-real approaches offer a way to create the solution in a simulation environment and apply it to the real-world problem, which is a safer and more economical way than the traditional trial-and-error approaches. Furthermore, some works focused on extraction of domain invariant features [592] to transfer knowledge across domains. Other works proposed learning by imitation/demonstrations approaches to help robots to learn manipulation skills [593].

### 4.10. Information Systems

Deep learning has received increasing attention in information systems. Major applications include social network analysis, information retrieval, and recommendation.

#### 4.10.1. Social Network Analysis

Social network analysis is an important problem in data mining. It targets social media networks such as Facebook, Twitter and Instagram to analyze their patterns and infer knowledge from them. Network representation learning is an important task in social network analysis. It encodes network data into low-dimensional representations, namely network embeddings, which effectively preserves network topology and other attribute information, facilitating subsequent tasks such as classification [594], link prediction [595], semantic evaluation [596], anomaly detection [597], and clustering [598].

Semantic evaluation helps machines understand the semantic meaning of users’ posts in social networks and infer the users’ opinions. Examples of sentiment classification are the SemEval [599] and Amazon purchase review [596]. Link prediction is widely used in recommendation and social ties prediction applications, where deep learning models are trained to learn robust representations to enhance prediction performance and deal with the scalability issue [595]. Popular models for link prediction include RBMs, DBNs [600], and GNNs [601]. In some studies, transfer learning with pretrained models (e.g., RBMs) was applied to improve the training efficiency and address the insufficient data issue [600]. Anomaly detection aims at spotting malicious activities in social networks, such as spamming and fraud. Such activities can be interpreted as outliers that deviate from the majority of normal activities. Deep learning approaches based on network embedding techniques are receiving increasing attention in this field [597]. Anomaly detection is also related to crisis response [602] which focuses on detecting natural and man-made disasters, where deep learning models are trained to identify information from the posts and classify them into classes such as bushfire and earthquake. It is worth noting that attention mechanisms have been widely adopted in sequence-based tasks to allow the deep learning models to focus on relevant parts of the input during the learning process. Attention layers are also used for aggregating important features from the local neighbors of nodes, as in graph attention networks [136].

#### 4.10.2. Information Retrieval

Deep learning approaches are also employed in document retrieval and web search applications [603]. A representative work is the deep-structured semantic modeling (DSSM) [603] which adopts a DNN for latent semantic analysis. A following work improved DSSM by applying convolutional layers to integrate representations extracted from each word in the sequence in order to generate representations for a subset of words [604]. Moreover, deep stacking networks were proposed for general information retrieval tasks, where multiple network blocks were stacked on top of each other to extract high-level, low-dimensional abstractions in the final feature space.

#### 4.10.3. Recommendation Systems and Others

Recommender systems play an important role in online shopping services by helping users discover items of interest from a large resource collection. A memory augmented graph neural network (MA-GNN) can capture both the long- and short-term user interests. Ma et al. [605] proposed memory augmented graph neural networks for sequential recommendation. Specifically, a GNN was used to model the item contextual information within a short-term period, a shared memory network was designed to capture the long-range dependencies between items, and co-occurrence patterns of related items were captured to model the user interests. Furthermore, a heterogeneous information network containing different types of nodes and links is a powerful information model in this field. Hence, researchers have proposed embedding methods to represent the network for recommender systems [606]. Other applications include bibliometric analysis such as citation prediction [607] and co-authorship network analysis. In such works, deep learning models, especially graph neural networks, were proposed to learn patterns from the citation networks, co-authorship networks, and heterogeneous bibliometric networks [608].

### 4.11. Other Applications

#### 4.11.1. Deep Learning in Food

Deep learning has recently been introduced in food science and engineering and has proved to be an advanced technology. The research of deep learning in food mainly focuses on the following topics.

(1) *Food Recognition and Classification.* Food analysis is important for the health of human beings. As image sensing has become an easy and low-cost information acquisition tool, food analysis based on images of food has become popular. Food images contain important information of food characteristics, which can be used to recognize and classify food to help people record their daily diets. Currently, with the great success of CNN in various recognition and classification tasks, several CNN variants have been adopted for food recognition and classification [609,610,611]. These methods achieve relatively good results, yet there is still room for improvement in accuracy and efficiency.
(2) *Food Calorie Estimation.* Food calorie estimation is widely adopted in many mobile apps to help people monitor and control nutrition intake, lose weight, and improve dietary habits to stay healthy. An image-based food calorie estimation method has been proposed and become popular [612]. It uses a multitask CNN and outperforms the traditional search-based methods. Following this, more CNN-based methods have been proposed for this task and proved that CNNs are effective for image-based food calorie estimation [613,614].
(3) *Food Quality Detection.* Food quality is vital for the health of human beings. Food quality detection can be further divided into subtopics of vegetable quality detection, fruit quality detection, and meat and aquatic quality detection. Among them, vegetables and fruits quality detection are currently hot and challenging topics. Stacked sparse AE and CNN were adopted for detecting vegetable quality based on hyperspectral imaging [615], where the diversity of surface defects in size and color are problematic for traditional methods based on the average spectrum of the whole sample. DNNs coupled with spectral sensing methods have been proposed for addressing problems of varieties classification, nutrient content prediction, and disease and damage detection in fruit quality detection [616,617].
(4) *Food Contamination.* Food contamination is a serious threat to human health, and thus has received great attention from all over the world. Several deep learning based methods have been proposed for predicting, monitoring, and identifying food contamination. For example, Song et al. [618] proposed using DNNs to predict the morbidity of gastrointestinal infections by food contamination. Gorji et al. [619] proposed using deep learning to automatically identify fecal contamination on meat carcasses. We refer to the survey paper [620] for more works and discussions. Generally, CNNs and their variants are still the most widely used and effective methods in this field.

#### 4.11.2. Deep Learning in Agriculture

Since the concept of precision farming was proposed, it has brought new problems and challenges. Deep learning has been adopted to develop agricultural intelligent machinery equipment due to its strong ability of extracting features from image and structured data.

(1) *Plant Diseases Detection.* Detecting diseases of crop is important for improving productivity. There are many types of disease species to be inspected. Deep learning technologies have been applied to crop disease classification or detection. For example, Ha et al. [621] proposed a deep learning based method to detect radish disease, where the radish was classified into diseased and healthy through a CNN. Ma et al. [622] also proposed using a CNN to recognize the four types of cucumber diseases. Lu et al. [623] proposed using CNNs to identify ten types of rice diseases, which proved the superiority of CNN-based methods in identifying rice diseases.
(2) *Smart Animal Breeding Environment.* Deep learning technologies have been adopted for monitoring and improving animal breeding environment. The currently most popular research in this domain is face recognition and behavior analysis of pigs and cows. For example, Yang et al. [624] proposed using a CNN combined with spatial and temporal information to detect nursing behaviors in a pig farm. Qiao et al. [625] proposed using a Mask R-CNN to settle cattle contour extraction and instance segmentation in a sophisticated feedlot surrounding. These works demonstrated the effectiveness of CNNs in automatic recognition of nursing interactions for animal farms. In addition, Hansen et al. [626] proposed a CNN-based method to recognize pigs. Tian et al. [627] proposed using CNN to count pigs.
(3) *Land Cover Change Detection.* Land cover change is vital for the natural basis of human survival, the Earth’s biochemical circle, and the energy and material circulation of the Earth system. One of the fundamental tasks in land cover change is cover classification. Deep learning techniques have been adopted for addressing this task. For example, Kussul et al. [628] proposed a multilevel deep learning architecture to classify the land cover and crop types using remote sensing data. Gaetano et al. [629] proposed a two-branch CNN for land cover classification. In addition, several CNN variants and transfer learning are adopted in the literature to validate land cover and classify wetland classes. See the survey papers [630,631] for details.

#### 4.11.3. Deep Learning in Chemistry

Deep learning has been actively and widely used in computational chemistry in the past few years. Several hot and popular research topics are discussed as follows. To build a molecule with a particular property would first require developing methods to accurately correlate any given structure to the property. These can then be used to intelligently design a molecule that maximizes the desired property. The final step is to design an efficient synthesis from readily available starting materials.

(1) *Materials Design.* Advanced materials are fundamental for many modern technologies such as batteries and renewable energy. Deep learning in this field is comparatively new, but there has been a rapid growth in the past few years. Xie et al. [632] proposed using a crystal CGNN to capture the crystalline structure for accurate and interpretable prediction of material properties. In addition, CGNNs and several CGNN variants have been proposed to predict the properties of bulk materials [633], optimize polymer properties [634], and explore chemical materials space [635]. These works demonstrated great potential of deep learning in exploring properties of materials. In addition to this, deep learning has been used to optimize synthesis parameters [636] and perform defect detection [637].
(2) *Drug Design.* Drug design is one of the most important applications of chemistry. Its aim is to identify molecules that achieve a particular biological function with maximum efficacy. Deep learning has been used to optimize the properties of molecules to improve potency and specificity, while decrease side effects and production costs. Specifically, AEs, GANs, and RNNs have been used to generate potent drug molecules [638,639,640]. More deep learning based methods are reviewed and discussed in recent papers [641,642,643].
(3) *Retrosynthesis.* The underlying challenge of retrosynthesis is similar to that of board games such as Chess and Go [644]. It can be solved by formulating the retrosynthesis as a tree search, where the branching factor is how many possible steps can be taken from a particular point. Therefore, inspired by AlphaGo, one of the predominant retrosynthetic AI was proposed by Segler et al. [645], which adopted the AlphaGo methodology of Monte Carlo Tree Search with deep neural network. This method has shown great potential. However, assessing synthesis plans is a challenging task. Other research has been using RNNs and AE to perform retrosynthetic analysis of small molecules [646].
(4) *Reaction Prediction.* Reaction prediction refers to taking a set of known reagents and conditions and predicting the products that will form. Deep learning has been used in this field to reduce the high computational cost in chemical space exploration. A representative work using DNNs to predict which products can be formed is presented by Wei et al. [647]. RNN variants and Siamese architectures have also been proposed for reaction prediction [648,649]. Emphasizing interpretability by using GCNN to predict reaction in a manner similar to human intuition is currently a hot research direction in this field [650].

## 5. Deep Learning Challenges and Future Directions

### 5.1. Efficiency

One of the growing problems of deep learning is computing efficiency. With the increasing volume of data and increasing complexity of DNNs, the requirement for computing power is increasingly high. This can be solved to some extent by advanced multicore GPUs, and tensor processing units (TPUs). However, more efficiency is often needed when optimizing deep learning architectures for embedded devices applications. This can be achieved through codesigning model architectures, training algorithms, software, and hardware to allow multimachine parallelism and scalable distributed deep learning [278]. For example, using compression techniques to compress the layers and thus optimize the model architecture; trimming the number of parameters to achieve a smaller footprint or a more efficient model; designing layers and architectures specifically with efficiency to save the number of parameters and avoid over-parameterization. Another challenging and promising direction is to design programmable computational arrays, bare-hardware implementation, and stochastic computation mechanisms [1].

### 5.2. Explainability

A major problem that affects the deployment of deep learning in various areas is the lack of transparency, which also called the “black box” problem. Deep learning algorithms learn from data to find patterns and correlations that human experts would not normally notice, and their decision-making processes often confuse even the engineers who created them. This might not be a problem when deep learning is performing a trivial task where a wrong result will cause little or no damage. However, when it comes to medical diagnosis or financial trades, a mistake can have very serious consequences. Therefore, the transparency issue is a potential liability when applying deep learning. Various visual analysis tools have been proposed to dissect DNNs and reveal what they actually learn, as investigated in the paper [651]. In addition, there are some techniques such as LIME [652] and Deep Lift [653] that can be used to explain the model using feature importance. However, the transparency issue has been well solved now. A promising way is to link neural networks to the existing well-known physical or biological phenomena [1]. This will help to develop a metaphysical relationship to demystify the DNN’s “brain”.

### 5.3. Generalizability

Generalizability is an important concern when applying a trained deep learning model in practice. It is challenging to demonstrate a deep learning model’s generalizability before implementing the model. To address the problem of model generalizability, many researchers try to use as much and as diverse data as possible to train a deep learning model. However, this is very challenging for some applications such as clinical scenarios where obtaining sufficient training samples with labels is extremely expensive and labor-intensive. Some researchers work on optimization algorithms that minimize the training error to achieve generalization. For example, Neyshabur et al. [654] proposed Path-SGD for better generalization, which is invariant to rescaling of weights. Hardt et al. [655] proposed to use stochastic gradient descent to ensure uniform stability, and thus to improve generalization for convex objectives. However, these are based on the assumption of having a “closed set” where the possible conditions in the test data are exactly the same as those in the training data. For many practical applications, the scenario is that “incomplete knowledge of the world is present at training time, and unknown classes can be submitted to an algorithm during testing” [656]. Therefore, a possible and promising research direction is using more generalizable or “open set” approaches to develop and evaluate deep learning models, such as open-set recognition [657].

### 5.4. Ethical and Legal Issues

Though deep learning has been widely deployed in many fields and has gained great success, some ethical and legal issues are emerging. There are two prominent issues. The first is the biased learning issue, where the model will provide a biased and prejudiced prediction/recommendation. One typical real-life example is the COMPAS (Correctional Offender Management Profiling for Alternative Sanctions) algorithm, which is used in US court systems to predict the probability that a defendant would become a recidivist [658]. This algorithm produced two times as many false positives for recidivism for black criminals (45%) than white criminals (23%). Another critical issue is the privacy of the deep learning training data. For example, social network face images could be used for training the deep learning model without the prior consent of the subjects. The lack of relevant governing frameworks on the regulation of these ethical and legal issues affects the wide application of deep learning in sensitive areas such as healthcare, finance, security, and law enforcement. The community is in desperate need of developing a relevant code of ethics and legal frameworks for addressing those issues.

### 5.5. Automated Learning

Deep learning has achieved great success in automatically learning representative features and performing recognition of these learned features. Although this has greatly eliminated the cumbersome process of handcrafting features, the development of deep learning models is resource-intensive, requiring significant domain knowledge and time to produce and compare dozens of models. Various software tool kits have been developed for getting production-ready deep learning models with great ease and efficiency [659,660]. However, these are not satisfactory enough for developing high-level and user-friendly platforms that are easy also for non-experts to adopt existing DNNs or to design their own solutions. Automated machine learning (AutoML) is a research field for this purpose. For deep learning, a variety of neural architecture search (NAS) methods have been proposed to automate the network designing process [291,661], which will be a promising way to solve the automated learning problem.

### 5.6. Distributed Learning

With the development of IoT and smart-world applications, massive numbers of smart mobiles and embedded devices are incorporated into the computing, resulting in network congestion and latency. Recent research in edge computing and in-device computing has provided solutions to this problem by utilizing IoT devices and some novel mechanisms within centralized and distributed computing frameworks [662,663]. Despite the achievements, several critical issues have yet to be well solved, and significant work still needs to be done. For example, the training of deep learning models in IoT devices is a problem that needs to be further solved. A possible way is to locally train the distributed and partial neural network input in IoT devices through offloading pretrained feature output for additional training at higher layers. In addition, developing appropriate paradigms to analyze data in a timely manner is another challenging problem requiring further research. Possible and promising research can be undertaken in the following directions: (1) distributed deep learning at the network edge, and more specifically, developing and optimizing parallel simultaneous edge network architectures for self-organization and runtime; and (2) in-device deep learning, and more specifically, implementing deep networks in IoT devices by considering the limited hardware and computational capabilities.

### 5.7. Privacy-Preserving Federated Learning

Nowadays, increasing privacy concerns have emerged along with the aggregation of distributed computing results. Privacy-preserving federated learning has become a solution for privacy-preserving deep learning [208,664]. By training deep learning models on separate datasets that are distributed across different devices or parties, it can preserve the local data privacy to a certain extent. However, despite the achievements, the challenge of protecting data privacy while maintaining the data utility through deep learning still remains. Potential and promising research problems and directions are: (1) how to effectively apply the privacy-preserving mechanisms [665,666] to federated learning frameworks for better privacy preservation; (2) develop efficient solutions to defend the final model against inference attacks extracting sensitive information from it; and (3) how to efficiently handle data memorization in federated learning to prevent privacy leakage.

### 5.8. Multimodal Learning

With the development of various sensor system, increasing numbers of data modalities can be obtained. Different modalities are characterized by different statistical properties, and thus it is important to discover the relationship between different modalities. Research in many application areas needs to be based on data from multiple modalities to achieve a more complete picture of the task, for example, biomedical studies typically involve both image and “omics” data. Therefore, multimodal learning, which can represent the joint representations of different modalities, is required for taking full advantage of all available data in such studies. This has been well recognized in several works [667,668] but deserves more attention. Potential research problems and directions are: (1) designing new learning frameworks with more powerful computing architectures to effectively learn feature structures of the multimodal data of increasing volume; (2) developing new deep learning models for multimodal data that take semantic relationships into consideration to mine the intermodality and crossmodality knowledge; and (3) designing online and incremental multimodal deep learning models for data fusion to learn new knowledge from new data without much loss of historical knowledge.

## 6. Conclusions

Deep learning has become a predominant method for solving data analysis problems in virtually all fields of science and engineering. The increasing complexity and the large volume of data collected by diverse sensor systems have brought about a significant development of deep learning, which has also fundamentally transformed the way data are acquired, processed, analyzed, and interpreted. In this paper we have provided a comprehensive investigation of deep learning in diverse sensor systems, starting from the fundamentals of deep learning models and methods, to mapping specific deep learning methods with individual suitable sensor systems. This paper also provides a comprehensive summary of implementation tips and links to tutorials, open-sourced codes, and pretrained models for new deep learning practitioners and those seeking to innovate deep learning in diverse sensor systems. In addition, this paper provides insights into research topics where deep learning has not yet been well-developed, but may have potential, and highlights the challenges and future of deep learning in diverse sensor systems. We hope this survey will provide an excellent self-contained and comprehensive reference for industry practitioners and researchers in the field.

## Figures and Tables

**Table 1:** Ten types of nonlinear activation functions.

Name	Definition
Sigmoid	$f\left(x\right)=\frac{1}{1+e^{-x}}$
Tanh	$f\left(x\right)=\frac{e^{x}-e^{-x}}{e^{x}+e^{-x}}$
ReLU	f(x)=max(0,x)
LeakyReLU	f(x)=max(0.1x,x)
Parametric ReLU	f(x)=max(ax,x)
ELU	$\left\{\begin{array}{ccc} x & \mathrm{for} & x\geq 0\\ \alpha (e^{x}-1) & \mathrm{for} & x<0 \end{array}\right.$
Probability	$\mathrm{sig}\left(x\right)=\frac{1}{1+e^{-x}}$
Softmax	$\mathrm{softmax}\left(x_{i}\right)=\frac{\exp(x_{i})}{\sum_{j}\exp\left(x_{j}\right)}$
Swish	$\sigma \left(x\right)=\frac{x}{1+e^{-x}}$
GELU	$f\left(x\right)=0.5x\left(1+\tanh\left[\sqrt{\frac{2}{\pi }}\left(x+ax^{3}\right)\right]\right)$ with a=0.044715

**Table 2:** Summary of popular CNN architectures.

Model	Usage	Main Contribution	Code	Year
AlexNet [37]	Recognition	Depth is essential	✓	2012
VGG [38]	Recognition	Small kernel size	✓	2013
GoogLeNet/Inception [39]	Recognition	Inception module (sparse connections)	✓	2013
ZfNet [40]	Visualisation	Understanding network activity	✓	2014
ResNet [41]	Recognition	Residual module (skip connections)	✓	2015
DenseNet [42]	Recognition	Dense concatenation	✓	2017
UNet [43]	Segmentation	U-shaped encoder-decoder architecture	✓	2015
Faster R-CNN [44]	Segmentation	Region proposal network	✓	2015
Highway Networks [45]	Recognition	Cross-layer connection	✓	2015
YOLO [46]	Detection	High efficiency ‘only look once’	✓	2016
Mask R-CNN [47]	Segmentation	Object mask	✓	2017
MobileNet [48]	Recognition/Detection	Depthwise separable convolutions	✓	2017
Pyramidal Net [49]	Recognition	Pyramidal structure	✓	2017
Xception [50]	Recognition	Extreme version of Inception	✓	2017
Inception-ResNet [51]	Recognition	Inception with residual connections	✓	2017
PolyNet [52]	Training solution	Optimize networks	✓	2017

**Table 3:** Databases for biometric applications.

Modality	Database
Face	Labeled Faces in the Wild http://vis-www.cs.umass.edu/lfw/ (accessed on 2 November 2022)
Face	YouTube Faces http://www.cs.tau.ac.il/wolf/ytfaces/ (accessed on 2 November 2022)
Face	AR Face database [387]
Face	MORPH https://uncw.edu/oic/tech/morph.html (accessed on 2 November 2022)
Iris	VSSIRIS https://tsapps.nist.gov/BDbC/Search/Details/541 (accessed on 2 November 2022)
Iris	Mobile Iris Challenge Evaluation http://biplab.unisa.it/MICHE/ (accessed on 2 November 2022)
Iris	Q-FIRE [388]
Iris	LG2200 and LG4000 https://cvrl.nd.edu/projects/data/ (accessed on 2 November 2022)
Fingerprint	FVC-onGoing https://biolab.csr.unibo.it/FVCOnGoing/UI/Form/Home.aspx (accessed on 2 November 2022)
Fingerprint	NIST SD27 https://www.nist.gov/itl/iad/image-group/nist-special-database-2727a (accessed on 2 November 2022)
Palmprint	PolyU Palmprint database http://www4.comp.polyu.edu.hk/csajaykr/database.php (accessed on 2 November 2022)
Voice	Google Audioset https://research.google.com/audioset/ (accessed on 2 November 2022)
Voice	VoxCeleb https://www.robots.ox.ac.uk/vgg/data/voxceleb/ (accessed on 2 November 2022)
Signature	GPDS-960 corpus https://figshare.com/articles/dataset/GPDS960signature_database/1287360/1 (accessed on 2 November 2022)
Signature	Signature verification competition 2004 [389]
Gait	CASIA-B http://www.cbsr.ia.ac.cn/english/Gait20Databases.asp (accessed on 2 November 2022)
Gait	OU-ISIR LP dataset http://www.am.sanken.osaka-u.ac.jp/BiometricDB/GaitLPBag.html (accessed on 2 November 2022)
Keystroke	CMU Benchmark Dataset https://www.cs.cmu.edu/keystroke/ (accessed on 2 November 2022)
EEG	EEG Motor Movement/Imagery Dataset https://physionet.org/content/eegmmidb/1.0.0/ (accessed on 2 November 2022)
EEG	BED [390]
ECG	ECG-ID https://physionet.org/content/ecgiddb/1.0.0/ (accessed on 2 November 2022)
ECG	PTB https://www.physionet.org/content/ptbdb/1.0.0/ (accessed on 2 November 2022)

**Table 4:** Databases for remote sensing applications.

Database	Task	Imagery	Resolution	Channels
UCMerced LandUse [436]	Image classification	Multispectral	-	115
University of Pavia [437]		Hyperspectral	1.3 m	11
Salinas [437]		Hyperspectral	3.7 m	224
WHU RS19 [438]	Scene classification	Aerial	up to 0.5 m	3
AID [439]		Aerial	-	3
NaSC-TG2 [440]		Multispectral	100 m	4
NWPU-RESISC45 [403]		Multispectral	30–0.2 m	3
NWPU VHR-10 [441]	Object detection	-	0.5–2 m	3
UCAS-AOD [416]		Aerial	-	3
HRSC2016 [442]		-	2–0.4 m	3
DOTA [443]		Aerial	-	3
DIOR [444]		Aerial	-	3
HRSID [445]		SAR	0.5–3 m	-

**Table 5:** Benchmarks for cybersecurity intrusion detection.

Dataset	Year	Main Attack Types
AWID3 [488]	2021	Flooding, injection, Botnet
CIC-IDS2017 [489]	2017	DoS/DDoS, port scan, web attacks
AWID2 [490]	2016	Flooding, injection, web attack
UNSW-NB15 [491]	2015	DoS, worms, back-doors, generic
ADFA-LD [492]	2013	Password, web attacks
NSL-KDD [449]	2009	DoS, Probe, U2R, R2L

## Data Availability

Not applicable.
